# Subclinical cardiac dysfunction may impact on fluid and vasopressor administration during early resuscitation of septic shock

**DOI:** 10.1186/s44158-023-00117-3

**Published:** 2023-08-28

**Authors:** Francesco Murgolo, Rossella di Mussi, Antonio Messina, Luigi Pisani, Lidia Dalfino, Antonio Civita, Monica Stufano, Altamura Gianluca, Francesco Staffieri, Nicola Bartolomeo, Savino Spadaro, Nicola Brienza, Salvatore Grasso

**Affiliations:** 1 Department of Precision-Regenerative Medicine and Jonic Area (DiMePRe-J), Section of Anesthesiology and Intensive Care Medicine, University of Bari “Aldo Moro”, Bari, Italy; 2 Department of Biomedical Sciences, IRCCS Humanitas Research Hospital, Humanitas University, Pieve Emanuele-Milano, Italy; 3 Interdisciplinary department of medicine, University of Bari, Bari, Italy; 4 Department of translation medicine, University of Ferrara, Ferrara, Italy

**Keywords:** Surviving Sepsis Campaign, Septic shock, Sepsis-related cardiac dysfunction, Hemodynamic resuscitation, Trans-pulmonary thermodilution

## Abstract

**Background:**

According to the Surviving Sepsis Campaign (SSC) fluids and vasopressors are the mainstays of early resuscitation of septic shock while inotropes are indicated in case of tissue hypoperfusion refractory to fluids and vasopressors, suggesting severe cardiac dysfunction. However, septic cardiac disfunction encompasses a large spectrum of severities and may remain “subclinical” during early resuscitation. We hypothesized that “subclinical” cardiac dysfunction may nevertheless influence fluid and vasopressor administration during early resuscitation. We retrospectively reviewed prospectically collected data on fluids and vasoconstrictors administered outside the ICU in patients with septic shock resuscitated according to the SSC guidelines that had reached hemodynamic stability without the use of inotropes. All the patients were submitted to transpulmonary thermodilution (TPTD) hemodynamic monitoring at ICU entry. Subclinical cardiac dysfunction was defined as a TPTD-derived cardiac function index (CFI) ≤ 4.5 min^−1^.

**Results:**

At ICU admission, subclinical cardiac dysfunction was present in 17/40 patients (42%; CFI 3.6 ± 0.7 min^−1^ vs 6.6 ± 1.9 min^−1^; *p* < 0.01). Compared with patients with normal CFI, these patients had been resuscitate with more fluids (crystalloids 57 ± 10 vs 47 ± 9 ml/kg PBW; *p* < 0.01) and vasopressors (norepinephrine 0.65 ± 0.25 vs 0.43 ± 0.29 mcg/kg/min; *p* < 0.05). At ICU admission these patients had lower cardiac index (2.2 ± 0.6 vs 3.6 ± 0.9 L/min/m^2^, *p* < 0.01) and higher systemic vascular resistances (2721 ± 860 vs 1532 ± 480 dyn*s*cm^−5^/m^2^, *p* < 0.01).

**Conclusions:**

In patients with septic shock resuscitated according to the SSC, we found that subclinical cardiac dysfunction may influence the approach to fluids and vasopressor administration during early resuscitation. Our data support the implementation of early, bedside assessment of cardiac function during early resuscitation of septic shock.

**Supplementary Information:**

The online version contains supplementary material available at 10.1186/s44158-023-00117-3.

## Background

According to the Surviving Sepsis Campaign (SSC), fluids and vasopressors are the mainstays of early resuscitation in patients with septic shock whereas inotropes are reserved for treating tissue hypoperfusion refractory to fluids and vasopressors, presumably due to severe septic cardiac dysfunction [1–3]. However, cardiac involvement during sepsis could not cause overt tissue hypoperfusion and therefore remain undiagnosed and untreated [3–5]. We reasoned that “subclinical” cardiac dysfunction could nevertheless have an impact on fluids and vasoconstrictors administration during early resuscitation.

According to recent expert opinion and guidelines, advanced hemodynamic monitoring should be warranted in patients with septic shock after early resuscitation [1, 2, 6]. Transpulmonary thermodilution (TPTD) is a minimally invasive hemodynamic monitoring technique that provides cardiac output (CO) and several CO-derived variables [7, 8], such as the global volume of the four cardiac chambers at end-diastole (GEDV), an estimate of cardiac preload [9, 10], the extravascular lung water (EVLW), an estimate of the amount of pulmonary edema [11, 12] and the cardiac function index (CFI). The latter has been validated in critically ill patients as a reliable estimate of left ventricular function, both against trans-cardiac thermodilution [13] and the “gold standard” echocardiographic method (both transesophageal and transthoracic) [14, 15].

In this study, we report the TPTD hemodynamic profile of consecutive patients with septic shock not treated with inotropes during early resuscitation and reviewed fluids and vasopressors administration during early resuscitation. Our hypothesis was that “subclinical” cardiac dysfunction (as identified by CFI) could impact on fluids and vasopressor administration during early resuscitation.

## Methods

We reviewed prospectically collected data on the initial resuscitation process outside the ICU and the TPTD profile at ICU admission of consecutive patients admitted to our ICU for septic shock, between March 2018 and May 2019. Septic shock was diagnosed according to the Sepsis-3 criteria [16]. The Independent Ethical Committee of the Azienda Ospedaliero-Universitaria Policlinico di Bari (Bari, Italy) approved the study (approval number: 7212; February 9, 2022). Written informed consent for the retrospective use of anonymous aggregate data was obtained from each patient or legal representative at the time of ICU admission.

We retrospectively selected the records of patients that (1) had been resuscitated outside the ICU without the use of inotropes according to our institutional protocol for early recognition and treatment of septic shock, strictly adherent to the SSC [1 and Online Supplement] and admitted to our ICU within 6 hours from the beginning of resuscitation; (2) were hemodynamically stable after initial resuscitation (i.e., all the following conditions: MAP > 65 mmHg, absence of severe arrhythmias, absence of skin mottling, capillary refill time lower than 3 s, urinary output > 0.3 ml/kg/h and a stable or decreasing trend of serum lactates [1, 17]); (3) were monitored for clinical reasons within 2 h from ICU entry with the TPTD technique (PiCCO Pulsion/Getinge, Medical Systems, Munich, Germany. Exclusion criteria were age lower than 18 years and pre-existing severe respiratory, cardiovascular, liver, and kidney diseases.

For each patient, we reviewed the administration of fluids and vasopressors during early resuscitation and the physiological data at baseline (i.e., when resuscitation started) and the first TPTD determination (i.e., within 2 h from the ICU admission).

At ICU admission the patients were ventilated with a tidal volume (VT) of 6–8 ml/kg PBW (predicted body weight), positive end-expiratory pressure (PEEP) and inspiratory oxygen fraction (FiO_2_) titrated according to the ARDS-net Low PEEP/FiO2 table and with a respiratory rate (RR) to keep arterial pH higher than 7.30 [18].

The TPTD technique has been described in detail elsewhere [7]. Briefly, it consists in the injection of a 10–20 ml bolus of cold (< 8 °C) isotonic saline through a central venous catheter. The thermodilution curve is recorded by a thermistor-equipped arterial catheter (usually introduced in the femoral artery) and CO is obtained curve through the Stewart-Hamilton principle. Intrathoracic volume (global end-diastolic volume (GEDV)) and extravascular lung water (EVLW)) are estimated by the mean transit time (MTt) and the exponential downslope time (DSt) of the thermodilution curve [10]. Briefly, CO*MTt is the intrathoracic thermal volume (ITTV) and CO*DSt is the pulmonary thermal volume (PTV) and, accordingly, the difference between ITTV and PTV represents the global blood volume contained at and-diastole in the four cardiac chambers (GEDV) [10, 19]. The TPTD-derived cardiac function index-CFI is expressed in min^−1^ [15]:

$$\mathrm{CFI}= \frac{\mathrm{CO}}{\mathrm{GEDV}}$$

In all the patients a central venous catheter was introduced in the superior vena cava through the right or the left internal jugular vein and the 5-F thermistor-tipped catheter (Pulsiocath PV2015L20A, Pulsion/Getinge Medical Systems, Munich Germany) was introduced in the right or left femoral artery (both percutaneously and with ultrasound guidance). TPTD determinations were obtained by injecting 20 ml of cold saline solution at temperature of < 8 °C through the distal port of the central venous catheter. Cardiac index (CI), central venous pressure (CVP) indexed systemic vascular resistances (SVRI) and pulmonary vascular permeability index (PVPI) were calculated through standard formulae [12, 14, 15]. Arterial and central venous oxygen gas analysis were recorded immediately prior the first TPTD determination to obtain arterial P/F ratio, central venous saturation (ScvO_2_), and central venous–arterial pCO_2_ difference (v-a) PCO_2_) [20].

### Statistical analysis

Continuous data are expressed as mean and standard deviation (SD) if normally distributed or as median and interquartile range (IQR) if not normally distributed. Normality of continuous data was tested through the Kolmogorov–Smirnov test. Categorical data are expressed as frequency and percentage.

Patients were divided into two group according to the CFI: cardiac dysfunction group, CFI ≤ 4.5 min^−1^ and normal cardiac function group, CFI > 4.5 min^−1^. Differences between the two groups were compared using Student’s *t* test, nonparametric Mann-Whitney *U* test and Fisher’s exact test, as appropriate. Each TPTD hemodynamic parameter was categorized as low, normal, and high according to previous studies [10] and the manufacturer specifications.

The 30-day overall survival (OS) was calculated through the Kaplan-Meier method and expressed as medians an 95% confidence interval. The survival curves were compared through the Log-rank test.

All tests of statistical significance were two-tailed and p-values less than 0.05 were considered statistically significant. Statistical analysis was performed using the SAS/STAT® Statistics, Version 9.4 (2013), SAS Institute Inc., Cary, NC, USA

## Results

**Fig. 1:**
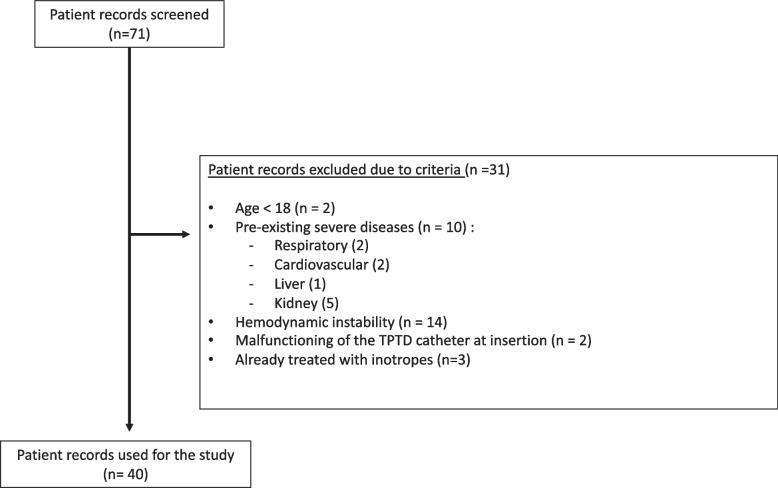
Flow diagram of patient records screened. Abbreviations: TPTD = trans-pulmonary thermodilution

Of the 71 consecutive patients admitted with a diagnosis of septic shock between March 2018 and May 2019, 31 were excluded due to exclusion criteria (Fig. 1), leading to a final study cohort of 40 patients. The CFI was ≤ 4.5 min^−1^ (3.6 ± 0.7 min^−1^) in 17 of them (42%, subclinical cardiac dysfunction group) and > 4.5 min^−1^ (6.6 ± 1.9 min^−1^) in the other 23 (58%, normal cardiac function group). Table 1 shows the baseline clinical and demographic characteristics. The 30-day mortality was not significantly different between the two groups (see Online Supplement).

**Table 1:** Demographical and clinical characteristics of the patients

	Cardiac dysfunction (CFI ≤ 4.5 min^−1^)	Normal cardiac function (CFI > 4.5 min^−1^)	*p* (*t* test)	*p* (exact Fisher-test)
Patients—no. (%)	17 (42)	23 (58)		
Age—year	57.5 ± 12.5	56.6 ± 11.6	0.680	
Male sex—no. (%)	11 (65)	13 (57)		0.747
Height—cm.	169.1 ± 11.1	170 ± 9.8	0.667	
Kg PBW	63.9 ± 10.8	65.6 ± 8.9	0.379	
BMI	27.6 ± 2.4	27.1 ± 4.6	0.440	
SAPS II	34 [30.8–37.8]	28 [24.5–37.5]	0.128	
Hemoglobin—g/dL	11.1 ± 3.0	11.2 ± 2.6	0.895	
Creatinine—mg/dL	1.3 [0.9–2.3]	1.1 [0.8–1.6]	0.208	
Bilirubin—mg/dL	0.8 [0.6–2.3]	0.8 [0.5–1.6]	0.722	
SOFA score	8 [8-10]	8 [6.5–9.5]	0.411	
Days before ICU admission	2.7 ± 1.5	3.2 ± 1.5		0.28
30 days mortality**—**no. (%)	12 (71)	9 (39)		0.062
Site of Infection—no. (%)				
Respiratory	9 (53)	14 (61)		0.898
Abdominal	4 (23)	5 (22)		
Genitourinary	1 (6)	0 (0)		
Bacteremia, site unspecified	3 (18)	4 (17)		

Data are expressed as as mean ± standard deviation or median [interquartile range], as appropriate

*Abbreviations*: *CFI* Cardiac function index, *PBW* Predicted body weight, calculated as follows: for men, 50 + 0.91 (height in centimeters 152.4); and for women, 45.5 + 0.91 (height in centimeters 152.4), *BMI* Body mass index, calculated as the weight in kilograms divided by the square of the height in meters, *SAPS II* Simplified Acute Physiology Score, *SOFA* Sequential Organ Failure Score

Table 2 reports the basic hemodynamic parameters at baseline (i.e., when resuscitation started) and the complete hemodynamic profile within 2 h from ICU admission. After early resuscitation, CI was 2.2 ± 0.6 L/min/m^2^ in the cardiac dysfunction group and 3.6 ± 0.9 L/min/m^2^ in the normal cardiac function group (*p* < 0.01), SVRI were 2721 ± 860 dyn*s*cm^−5^*m^2^ in the cardiac dysfunction group and 1532 ± 480 dyn*s*cm^−5^*m^2^ in the normal cardiac function group (*p* < 0.01) and ScvO_2_ was 72.9 ± 10.8% in the cardiac dysfunction group and 81.2 ± 7% in the normal cardiac function group (*p* < 0.01). The (a–v) PCO_2_ difference was significantly higher in the cardiac dysfunction group (5 ± 1.8 vs 3.4 ± 1 mmHg, *p* < 0.01).

**Table 2:** Hemodynamic parameters at baseline (start of resuscitation) and post-resuscitation, at the first TPTD determination

	Cardiac Dysfunction (CFI ≤ 4.5 min^–1^)		Normal Cardiac Function (CFI > 4.5 min^–1^)	
Patients – no. (%)	17 (42)		23 (58)	
	Baseline (resuscitation start)	Post-resuscitation (5–6 h from baseline)	Baseline (resuscitation start)	Post-resuscitation (5–6 h. from baseline)
CFI (min ^−1^)	–	3.6 ± 0.7^**#**^	–	6.6 ± 1.9 ^**#**^
CI (L/min/m^2^)	–	2.2 ± 0.6^**#**^	–	3.6 ± 0.9 ^**#**^
GEDVI (mL/m^2^)	–	609 ± 126^**#**^	–	562 ± 121^**#**^
SVRI (dyn_*_s_*_cm^−5^_*_m^2^)	–	2721 ± 860^**#**^	–	1532 ± 480 ^**#**^
EVLWI (ml/kg)	–	9.0 [8.0–11.6]	–	9.0 [7.3–11.0]
PVPI	–	2.2 ± 0,6^**#**^	–	2.4 ± 0.9 ^**#**^
HR (bpm)	98.5 ± 15.7^**#**^	89.9 ± 16.5^#*^	98.4 ± 20.1^**#**^	93.1 ± 18.5^**#**^
MAP (mmHg)	61.2 ± 4.4^**#**^	80.1 ± 6.4 ^*^	60.1 ± 3.7^**#**^	75 ± 5.5 ^*#^
CVP (mmHg)	–	11.1 ± 3.9^**#**^	–	10.4 ± 5.1^**#**^
PaO_2_ (mmHg)		109 ± 19	–	111 ± 19
FiO_2_ (%)		72 ± 18	–	72 ± 19
PaO_2_/FiO_2_		162 ± 60	–	161 ± 39
(v-a) PCO_2_ (mmHg)	–	5 ± 1.8	–	3.4 ± 1 ^**#**^
ScvO_2_ (%)	–	72.9 ± 10.8^**#**^	–	81.2 ± 7.0 ^**#**^
Lac (mmol/L)	4.5 [2.8–7.4]	4.4 [3.1–7.6]	3.7 [2.7–5.7]	3.0 [2.0–4.3]

Data are expressed as mean ± standard deviation or median [interquartile range], as appropriate

^*^ *p* < 0.05 versus baseline, within the same group

^#^*p* < 0.05 versus post-resuscitation, between the two groups

*Abbreviations*: *CFI* Cardiac Function Index, *CI* Cardiac index, *GEDVI* Global end-diastolic volume index, *SVRI* Indexed systemic vascular resistances, *EVLWI* Extra-vascular lung water index, *PVPI* Pulmonary vascular permeability index, *HR* Heart rate, *MAP* Mean arterial pressure, *PaO*_*2*_ Partial pressure of oxygen, *FiO*_*2*_ Inspiratory oxygen fraction, *(v - a) PCO*_*2*_ Central venous-arterial CO_2_ difference, *ScvO*_*2*_ Central venous oxygen saturation, *Lac* Arterial lactates, *CVP* Central venous pressure

Table 3 quantifies the therapeutical approach to fluids and vasopressors during early resuscitation in both groups. The cumulative dose of fluids (crystalloids in all the patients) was 57 ± 10 ml/kg PBW in the cardiac dysfunction group and 47 ± 9 ml/kg PBW in the normal cardiac function group (*p* < 0.01). The vasoconstrictors dose (norepinephrine in all the patients) was 0.65 ± 0.25 mcg/kg/min in the cardiac dysfunction group and 0.43 ± 0.29 mcg/kg/min in the normal cardiac function group (*p* < 0.01).

**Table 3:** Therapeutical approach to early resuscitation

Patients–no. (%)	Cardiac dysfunction (CFI ≤ 4.5 min^−1^) 17 (42)	Normal cardiac function (CFI > 4.5 min^−1^) 23 (58)
Crystalloids (mL/kg PBW)	57 ± 10	47 ± 9 ^**#**^
N. of patients treated with crystalloids	17	23
Norepinephrine (mcg/kg/min)	0.65 ± 0.25	0.43 ± 0.29 *
N. of patients treated with norepinephrine	17	23

Data are expressed as mean ± standard deviation or median [Interquartile range], as appropriate

^*^p < 0.05 compared to the cardiac dysfunction group

^**#**^*p* < 0.01 compared to the cardiac dysfunction group

*Abbreviations*: *CFI* Cardiac function index, *PBW* Predicted body weight

**Fig. 2:**
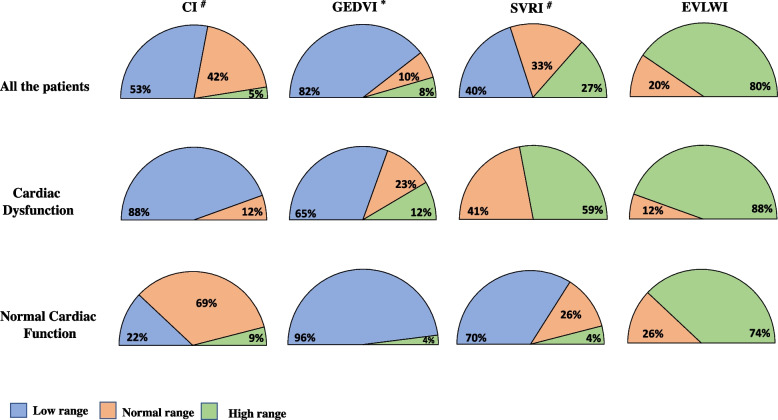
Patterns of distribution of “low”, “normal”, and “high” range of CI [3–5 mL/min/m^2^], GEDVI [680–800 mL/m^2^], SVRI [1700–2400 dyn_*_s_*_cm^−5^_*_m^2^], and EVLWI [3–7 mL/kg/m^2^] in the whole study population and partitioned by cardiac function groups. Abbreviations: CI = cardiac index; GEDVI = global end-diastolic volume index; SVRI = indexed systemic vascular resistances; EVLWI = extra-vascular lung water index

Figure 2 reports the patterns of CI, GEDVI, SVRI, and EVLWI. According to the Fisher Exact test, CI, GEDVI, and SVRI had different distribution patterns between the two groups (*p* < 0.01 for CI and SVRI and *p* = 0.047 for GEDVI).

## Discussion

We found that “subclinical” cardiac dysfunction at ICU admission was present in 42% of patients with septic shock fully resuscitated without the use of inotropes and that during early resuscitation these patients were treated with more fluids and vasopressors compared to patients with normal cardiac function.

According to the SSC guidelines, inotropes are second line agent during early resuscitation and their use is restricted to patients with signs of tissue hypoperfusion refractory to fluids and vasopressors, presumably attributable to severe septic cardiac dysfunction [1, 2]. As for study protocol, we included patients with septic shock that were admitted in ICU with no signs of tissue hypoperfusion and that were not administered inotropes during early resuscitation. Thus, our patients had reached sufficient hemodynamic stability with fluids and vasopressors, according to our hospital protocol for early recognition and treatment of septic shock that is strictly adherent to the SSC [1, Online Supplement]. However, we found in 42% of these patients a CFI below ≤ 4.5 min^−1^, indicating some degree of cardiac dysfunction, that we designated as “subclinical” since it remained undiagnosed and untreated during the early resuscitation process (we selected patients not treated with inotropes). The CFI is a rather reliable index of global left ventricular function. Ritter and coworkers compared TPTD and Pulmonary artery catheter (PAC) in 21 ICU patients (9 with septic shock) and found that CFI correlated with the left ventricular stroke work index [13]. Coombs and coworkers compared CFI and left ventricular fractional area of change (LVFAC) in 33 mechanically ventilated patients (16 with septic shock) and found a significant correlation (*r* = 0.87, *p* < 0.0001) [15]. Furthermore, Jabot and coworkers compared CFI with left ventricular ejection fraction (LVEF) obtained through transthoracic apical four and two-chamber views (deemed as the standard tool for measuring LVEF) in 39 patients (25 with septic shock) and,again, found a significant correlation (*r* = 0.67; *p* < 0.05) [14]. Since we found that even “subclinical” forms of cardiac dysfunction may impact on the administration of fluids and vasopressors during early resuscitation, one possible implication of our study is that cardiac function should be addressed very early when resuscitating a patient with septic shock. Since hemodynamic monitoring with TPTD is relatively invasive and time consuming and it cannot reveal the nature and type of cardiac dysfunction, the ideal tool in this context would be echocardiography. Indeed, Viellard-Baron and coworkers showed global left ventricular hypokinesia (left ventricular ejection fraction < 45% assessed through trans-esophageal echocardiography is frequent in adult septic shock) in 60% of a cohort of 67 patients with septic shock without previous global left ventricular hypokinesia) [5]. Of note, current guidelines suggest echocardiography during initial resuscitation only where available [1] whereas expert’s consensus suggests early use of echocardiography to guide initial fluid resuscitation, particularly in patients with clinical evidence of ventricular failure or persistent shock [6].

Confirming our study hypothesis, subclinical cardiac dysfunction impacted on fluids administration during early resuscitation. It is difficult to explain the mechanisms by which subclinical cardiac dysfunction may have influenced fluids administration during early resuscitation. Patients with “subclinical” cardiac dysfunction received more norepinephrine and their SVRI was in the higher range in 59% of the cases, whereas patients with normal cardiac function received less norepinephrine and only 4% of them had SVRI in higher range (Fig. 2). Several studies have highlighted the multifaceted effects of norepinephrine on cardiac function [21, 22]. In particular, besides increasing arteriolar resistances, norepinephrine decreases capillary permeability and induces an endogenous increase in venous return through venous vasoconstriction, improving cardiac output [23]. On the other hand, prolonged or excessive use of norepinephrine may cause coronary and digital ischemia affecting myocardial perfusion [24, 25], and may decrease global left ventricular contractility in patients with septic shock [5]. Recently Guarracino and colleagues demonstrated that norepinephrine by increasing the arterial elastance and worsening the arterial-ventricular coupling [21, 26], may impair cardiac output in patients with septic shock and decreased LV end-systolic elastance, a load-independent LV contractility parameter [3]. However, we must point out that we have no data to demonstrate any effect of norepinephrine on cardiac function in our patients and thus any effort to explain our findings remains purely speculative.

The SSC recommend volume expansion through a qualitative approach, based on frequent re-evaluation of clinical and physiological variables (skin mottling, capillary refilling time, temperature, urine output, respiratory rate, heart rate, arterial blood pressure), on the trend of plasma lactates and on the so-called dynamic indices of fluid responsiveness [1]. The latter include pulse pressure or stroke volume variation in response to passive leg raising [27] or fluid challenge [28]. In our patients we found hypovolemia at ICU admission in 96 % of the cases with normal cardiac function *versus* 65% of the cases with cardiac dysfunction (Fig. 2). This in our opinion may explain the fact that the EVLWI was similar in the two groups, despite patients with cardiac dysfunction received more fluids that patients with normal cardiac function (Table 3). We point out that, overall, our data suggest that establishing the “adequateness” of fluid replacement during early resuscitation may be difficult. Of note, ultrasound could be a useful tool to assess beside cardiac function the volemic status at bedside during early resuscitation [29, 30].

## Limitations

We must acknowledge some study limitations. (1) Our study was retrospective and thus we do not have physiological data to explain our findings. In particular, through trans-thoracic or trans-esophageal echocardiography at ICU admission it would have been possible to assess type and nature of the cardiac dysfunction and ventricular-arterial coupling; (2) we have no detailed data on the use of dynamic indices of preload to guide fluid resuscitation in our patients, but we point out that the use of these indices is strongly suggested in our institution guidelines for resuscitation of septic shock that are strictly adherent to the SSC; (3) our patients were not submitted to either transthoracic or trans-esophageal echocardiography during early resuscitation and, as discussed above, very early echocardiography would have been of great interest in to reveal subclinical cardiac dysfunction during initial resuscitation (4) our study was monocentric and hence local practice could have influenced the approach to initial resuscitation. On the other hand, our data may reflect the application of the SSC in the clinical context.

## Conclusions

We found that after early resuscitation outside the ICU, 42% of a cohort of 40 patients with septic shock that were hemodynamically stable after early resuscitation had a low CFI. Compared to patients with normal CFI, these patients received more fluids and vasopressors during early resuscitation and had a different hemodynamic profile. Overall, our study supports bedside assessment of cardiac function during initial resuscitation and advanced hemodynamic monitoring at ICU admission, even in patients that seem to respond to fluid and vasopressors.

### Supplementary Information

**Additional file 1.** Online supplement. Subclinical cardiac dysfunction may impact on fluid and vasopressor administration during early resuscitation of septic shock.

## Data Availability

The raw data supporting the conclusions of this article will be made available by the authors, without undue reservation.

## Abbreviations

SSC: Surviving sepsis campaign
TPTD: Trans-pulmonary thermodilution
CO: Cardiac output
GEDV: Global end-diastolic volume
EVLW: Extravascular lung water index
CFI: Cardiac function index
ICU: Intensive care unit
MAP: Mean arterial pressure
VT: Tidal volume
PBW: Predicted body weight
PEEP: Positive end-expiratory pressure
FiO_2_: Inspiratory oxygen fraction
MTt: Mean transit time
DSt: Downslope time
PTV: Pulmonary thermal volume
ITTV: Intrathoracic thermal volume
CI: Cardiac index
SVRI: Indexed systemic vascular resistances
PVPI: Pulmonary vascular permeability index
CVP: Central venous pressure
S_CV_O_2_: Central venous oxygen saturation
LV: Left ventricular
Ees: End-systolic elastance
Ea: Atrial elastance
VAC: Ventricular-arterial coupling
