# Supplementary material for: Subclinical cardiac dysfunction may impact on fluid and vasopressor administration during early resuscitation of septic shock
Source: J Anesth Analg Crit Care. 2023 Aug 28;3:29. doi: 10.1186/s44158-023-00117-3 (PMC10463881; doi:10.1186/s44158-023-00117-3)

ONLINE

SUPPLEMENT

Subclinical cardiac dysfunction may impact on fluid and vasopressor administration during early resuscitation of septic shock

Francesco Murgolo MD, Rossella di Mussi MD, Antonio Messin MD, Luigi Pisani MD PhD, Lidia Dalfino MD, Antonio Civita MD, Monica Stufano MD, Altamura Gianluca MD, Francesco Staffieri MD, PhD, Nicola Bartolomeo MD, PhD, Savino Spadaro MD, PhD, Nicola Brienza MD and Salvatore Grasso MD.

TABLE OF CONTENTS

1. Institutional protocol…………………………………………………………………..…3

2. Kaplan-Meier plot of the probability of survival from enrollment to day 30…………..…70

1. INSTITUTIONAL PROTOCOL

ALLEGATO N. 11
Alla delib. n. 0148/00 del 24 GEN 2020

Policlinico di Bari - Ospedale Giovanni XXIII	**Processo Operativo**<br>**PDTA per l'Identificazione Precoce e la Gestione Tempestiva della Sepsi nel paziente adulto**	**P.O IGSO Rev. 00**
**REGIONE PUGLIA**<br>**AZIENDA OSPEDALIERO- UNIVERSITARIA**<br>**CONSORZIALE POLICLINICO DI BARI**	**COPIA CONTROLLATA: ☒ SI n.° 1 ☐ No**	**Pag 1 di 10**

Rev	Data di emissione	Modifiche	**Redazione**	Firma
00	24 Gennaio 2020	Prima emissione	U.O.C Anestesia e Rianimazione Univ. II	F.to Prof. S. Grasso
00			U.O.C Ginecologia e Ostetricia	F.to Prof. E. Cicinelli
00			U.O.C Pronto Soccorso	F.to Dott. V. Procacci
00			**Approvazione**	
00			Direzione Sanitaria	F.to Dott.ssa M. Carlucci
00			**Verifica**	
00			UQA	F.to Dott. F. Lavalle
00			UGR	F.to Prof. A. Dell'Erba
00			**Emissione**	
00			UQA	F.to Dott. F. Lavalle

INDICE

1. Scopo e campo di applicazione pag 3
2. Riferimenti normativi e bibliografici pag 3
3. Termini e definizioni pag 5
4. Attività pag 7
5. Pianificazione pag. 8
6. Attività operativa pag. 8
   - 6.1 Premessa pag. 8
   - 6.2 Screening del rischio evolutivo e diagnosi precoce di sepsi e di shock settico in ospedale pag. 9
   - 6.3 Approccio immediato al paziente con sepsi grave/shock sospetti o documentati: il bundle Sepsis Six modificato pag. 12
   - 6.4 Percorso diagnostico-terapeutico-assistenziale dei pazienti con infezione o sepsi presunta/documentata in ospedale pag. 14
     - 6.4.1 Pronto Soccorso Centrale pag. 15
     - 6.4.2 Reparti di area medica e chirurgica pag. 19
     - 6.4.3 Pronto Soccorso Ostetrico e reparto di Ostetricia pag. 22
     - 6.4.4. Unità di Terapia Intensiva specialistiche e Reparti di rianimazione pag. 32
7. Verifica dei processi e delle prestazioni pag. 34
8. Azioni di miglioramento pag. 34
9. Identificazione, emissione e archiviazione dei documenti pag. 35
10. Redazione e revisione del documento e gruppo di lavoro pag. 36
11. Allegati
12. Allegato 1. Strumenti per lo screening della sepsi pag. 2
13. Allegato 2. Strumenti per la diagnosi di sepsi pag. 9
14. Allegato 3. Criteri di alto rischio di sepsi nella popolazione generale e nella popolazione ostetrica pag. 13
15. Allegato 4. Il bundle sepsis six modificato pag. 15

1. SCOPO E CAMPO DI APPLICAZIONE

Obiettivo del PDTA è la standardizzazione dell'approccio a tutti i pazienti adulti e alle pazienti ostetriche con infezione, sepsi o shock settico presunti o documentati, in un contesto di cure primarie (Pronto Soccorso centrale, UU.OO di area medica e chirurgica, Unità di Terapia Intensiva specialistiche e reparti di Rianimazione, Pronto Soccorso ostetrico, UU.OO di Ostetricia e Ginecologia, sala parto), al fine di garantirne la tempestiva identificazione e la corretta gestione.
Il Processo Operativo si applica a tutti i professionisti sanitari coinvolti nell'assistenza ai pazienti che afferiscano al Pronto Soccorso centrale o che siano degenti nelle suddette UU.OO di area medica o chirurgica o nelle Unità di Terapia Intensiva specialistiche e nei reparti di Rianimazione e alle pazienti in gravidanza/puerperio che afferiscano al Pronto Soccorso Ostetrico o siano ricoverate nelle UU.OO di Ostetricia e Ginecologia durante il percorso nascita.

2. RIFERIMENTI NORMATIVI E BIBLIOGRAFICI

- UNI EN ISO 9001: 2015.
- Fleischmann et al Assessment of Global Incidence and Mortality of Hospital-treated Sepsis Current Estimates and Limitations; on behalf of the International Forum of Acute Care Trialists. Am J Respir Crit Care Med, 2016; 259-272.
- Istituto Superiore di Sanità. Primo Rapporto ItOSS. Sorveglianza della mortalità materna. A cura di Stefania Dell'Oro, Alice Maraschini, Ilaria Lega, Paola D'Aloja, Silvia Andreozzi e Serena Donati. 2019, xiii, 69 p.
- World Health Organization. Global Maternal Sepsis Study. https://srhr.org/sepsis/.
- Say L, Chou D, Gemmill A et al. Global causes of maternal death: a WHO systematic analysis. Lancet Glob Health. 2014; 2: e323-33.
- Ministero della Salute. Near miss ostetrici in Italia: la sepsi, l'eclampsia, l'embolia di liquido amniotico e l'emoperitoneo spontaneo. https://www.epicentro.iss.it/itoss/pdf/12gen2018/D%27Aloja_convegnoITOSS_2018-def.pdf
- The Third International Consensus Definitions for Sepsis and Septic Shock (Sepsis-3). JAMA. 2016; 315(8): 801-810.
- Bonet M, Pileggi VN, Rijken MJ, et al. Towards a consensus definition of maternal sepsis: results of a systematic review and expert consultation. Reproductive Health, 2017; 14:67.
- Surviving sepsis campaign: International Guidelines for management of sepsis and septic shock: 2016. Crit Care, 2017.
- Levy MM, Evans LE and Rhodes A. The Surviving Sepsis Campaign Bundle: 2018 update. Intensive Care Med, 2018; 44:925-928.
- Bowyer L, Robinson HL, Barrett H. SOMANZ guidelines for the investigation and management sepsis in pregnancy. Aust N Z J Obstet Gynaecol 2017; 57: 540–551.

- Singer AJ. Quick SOFA Scores Predict Mortality in Adult Emergency Department Patients With and Without Suspected Infection http://dx.doi.org/10.1016/j.annemergmed.2016.10.007
- Julian M Williams et al. Systemic Inflammatory Response Syndrome, Quick Sequential Organ Function Assessment, and Organ Dysfunction: Insights From a Prospective Database of ED Patients With Infection Chest, 2017; 151: 586-596.
- Raith EP, Udy AA, Bailey M, et al. Prognostic Accuracy of the SOFA Score, SIRS Criteria, and qSOFA Score for In-Hospital Mortality Among Adults With Suspected Infection Admitted to the Intensive Care Unit. JAMA, 2017; 317:290-300.
- Sepsis: recognition, diagnosis and early Sepsis: recognition, diagnosis and early management management. NICE guideline Published: 13 July 2016 nice.org.uk/guidance/ng51.
- Umar A, Ameh CA, Muriithi F, Mathai M. Early warning systems in obstetrics: A systematic literature review. PLoS ONE, 2019; 14: e0217864. https://doi.org/10.1371/journal. pone.0217864.
- Umar A, Ameh CA, Muriithi F, Mathai M. Early warning systems in obstetrics: A systematic literature review. PLoS ONE, 2019; 14: e0217864. https://doi.org/10.1371/journal.pone.0217864.
- Singer t al. Diagnostic characteristics of a clinical screening tool in combination with measuring bedside lactate level in emergency department patients with suspected sepsis. 2014;21(8):853-7.
- Donati S. Dossier "La prevenzione e gestione della sepsi materna" in: Corso di Formazione a distanza: La prevenzione e gestione della sepsi materna – Italian Obstetric Surveillance System - Istituto Superiore di Sanità; ZADIG Editore - Ottobre 2018.
- Sager et al. Procalcitonin-guided diagnosis and antibiotic stewardship revisited BMC Medicine (2017) 15:15 DOI 10.1186/s12916-017-0795-7.
- Marik P, Bellomo R. A rational approach to fluid therapy in sepsis. BJA, 2016;116: 339–49.
- Genga K, Russell JA. Early liberal fluids for sepsis patients are harmful. Crit Care Med, 2016; 44(12):2258-2262.
- Leisman D, et al. Association of fluid resuscitation initiation within 30 minutes of severe sepsis and septic shock recognition with reduced mortality and length of stay. Ann Emerg Med. 2016;68:298-311.
- Marik PE. Fluid responsiveness and the six guiding principles of fluid resuscitation. Crit Care Med, 2016; 44 : 1920-22.
- Haydar SA, et al. Effect of bedside ultrasonography on the certainty of physician clinical decision making for septic patients in the emergency department. Ann Emerg Med, 2012;60:346-58.
- Nazerian P, Volpicelli G, Vanni S, et al. Accuracy of lung ultrasound for the diagnosis of consolidations when compared to chest computed tomography Am J Emerg Med. 2015 May;33(5):620-5
- AGENAS Agenzia Nazionale dei Servizi Sanitari Regionali. Linee di indirizzo clinico-organizzativo per la prevenzione delle complicanze in ostetricia 2017.
- ACOG Committee Opinion. Guidelines for Diagnostic imaging during pregnancy and lactation. Vol 130 (4), 2017.
- Royal College of Obstetricians & Gynaecologists. Green-top Guideline No. 64a, 1st edition, April 2012. Bacterial Sepsis in Pregnancy. https://www.rcog.org.uk/globalassets/documents/guidelines/gtg_64a.pdf.

3. TERMINI E DEFINIZIONI.

Sepsi: disfunzione d'organo a rischio-vita conseguente a una risposta de-regolata dell'organismo a un'infezione.

Sepsi materna: disfunzione d'organo a rischio-vita conseguente a una risposta de-regolata dell'organismo a un'infezione contratta durante la gravidanza, il parto e il periodo post-aborto o post-partum.

Shock settico: subset della sepsi in cui le alterazioni emodinamiche e cellulari-metaboliche sono di gravità tale da incrementare in misura sostanziale il rischio di morte.

Percorso nascita: comprende gravidanza, travaglio, parto e periodo post-partum o post-aborto.

Periodo post-partum o post-aborto: lasso di tempo fra la rottura delle membrane o il parto e il 42° giorno del puerperio.

ABBREVIAZIONE	DESCRIZIONE
ClCr	Clearance della creatinina
CTG	Cardiotocografia
CVC	Catetere venoso centrale
EGA	Emogasanalisi
FC	Frequenza cardiaca
FDA	*Food and Drug Administration*
FiO2	Frazione inspiratoria di O2
FR	Frequenza respiratoria
HIV	Human Immunodeficiency Virus
MDR	*Multi-drug resistant*
MEOWS	*Modified Early Obstetric Warning System*
mSOFA score	*Modified Sequential (Sepsis-related) Organ Failure Assessment score*

ABBREVIAZIONE	DESCRIZIONE
NEWS	*National Early Warning Score*
OMS	Organizzazione Mondiale della Sanità
PAM	Pressione arteriosa media
PaO2	Pressione arteriosa di O_2
PAS	Pressione arteriosa sistolica
PCT	Procalcitonina
PiCC	Catetere venoso centrale inserito per via periferica
POCUS	Point Of Care Ultrasonography
PPROM	Rottura prematura delle membrane nella gravidanza pretermine (< 37 settimane)
qSOFA	Quick SOFA
RMN	Risonanza Magnetica Nucleare
SO	Sala operatoria
SOFA score	*Sequential (Sepsis-related) Organ Failure Assessment score*
SpO_2	Saturazione periferica di Ossigeno
SSC	*Surviving Sepsis Campaign*
TAC	Tomografia assiale computerizzata
TC	Temperatura corporea
TI	Terapia Intensiva
UO/UU.OO	Unità Operativa/Unità Operative
US	Ultrasonografia

4. ATTIVITA'

	UU.OO.	Referenti Qualità UU.OO.	DS	Rischio Clinico	Responsabile Qualità Azienda	Direzione Strategica
**Redazione e Strutturazione del Processo**	Anestesia e Rianimazione II<br>Pronto soccorso<br>Ginecologia<br>Malattie Infettive<br>Igiene<br>UGR					
**Verifica dei contenuti**		x		x	x	
**Approvazione del Processo**			x			
**Adozione del Processo**						x
**Identificazione, Emissione ed archiviazione della Documentazione**					x	
**Monitoraggio dei Risultati Ottenuti ***	x	x	x	x	x	
**Attuazione del Miglioramento (Modifiche Processo)**	x	x	x	x	x	

5. PIANIFICAZIONE

Il presente PDTA sarà svolto, iso-risorse, nelle strutture afferenti al Processo Operativo e dalle figure professionali in esse presenti. In corso di applicazione del Processo Operativo potranno essere necessarie ulteriori risorse, organizzative ed economiche, che saranno concordate con la Direzione Sanitaria e con il CCIO.

6. ATTIVITÀ OPERATIVA

6.1 Premessa

La sepsi è una sindrome generata da una risposta de-regolata e abnorme dell'organismo a un'infezione, che si traduce in un danno d'organo.
Molto frequente e a rapida evoluzione, essa è gravata da elevata mortalità. A livello mondiale, l'incidenza annua di sepsi **nella popolazione generale** supera i 270 casi per 100.000 abitanti, con un tasso di più di 2 milioni di casi e una mortalità che nello shock settico può raggiungere il 40-70% dei casi.
L'incidenza della sindrome è purtroppo destinata ad aumentare, in primo luogo per l'estensione delle categorie a maggior rischio, legata all'allungamento della vita media e all'implementazione delle misure di supporto delle funzioni vitali nei pazienti critici e delle terapie immunosoppressive in trapiantologia, oncologia e reumatologia.
Poiché la sepsi può svilupparsi in risposta ad infezioni di frequente riscontro, come la polmonite, le infezioni del tratto genito-urinario e le infezioni intra-addominali e poiché tali infezioni possono sia essere causa di un accesso ospedaliero, che svilupparsi durante la degenza in ospedale, il *setting* assistenziale in cui la sepsi può insorgere è estremamente vasto e variegato.
Durante il **percorso nascita**, l'incidenza di sepsi è compresa tra 9 e 49 casi per 100.000 parti per anno, con una mortalità che oscilla tra il 2% e il 17.6% e che raggiunge il 30% dei casi nello shock settico. Quando l'esordio della sindrome passa misconosciuto e il deterioramento clinico rapidamente evolve in arresto cardiaco, la mortalità materna supera il 50%. Allarmante è il trend in ascesa dell'impatto della sepsi sulla sicurezza materna. In Italia, se negli anni 2006-2012 essa era quarta causa di morte materna diretta, negli anni 2013-2017 si è portata al secondo posto, dopo l'emorragia ostetrica.
Come dimostrato da oramai solide evidenze scientifiche riguardanti la popolazione generale, **la sepsi è una sindrome tempo-dipendente** in cui il rischio di morte è correlato alla rapidità con la quale vengono intrapresi percorsi diagnostico-terapeutici e assistenziali standardizzati e di comprovata efficacia.
Ancor più che nella popolazione generale, **nella popolazione ostetrica** il riconoscimento precoce e la gestione tempestiva della sindrome sono elementi chiave a fini prognostici. In questo *setting*, difatti, si verifica sia una fisiologica deviazione dalla normalità di alcuni parametri su cui nella popolazione generale si fonda la diagnosi di infezione e di sepsi, che una limitazione nel compenso alle alterazioni fisiopatologiche sepsi-indotte, incrementando in misura sostanziale il rischio di diagnosi tardiva, in una popolazione in cui il deterioramento clinico è più rapido.

Pertanto, la sepsi e lo shock settico sono condizioni cliniche che richiedono un approccio in emergenza e l'adesione agli standard di diagnosi e cura rappresenta una priorità per chi ha responsabilità di governo delle Aziende Sanitarie e per tutti i professionisti del settore.

6.2 Screening del rischio evolutivo e diagnosi precoce di sepsi e di shock settico in ospedale.

In accordo a quanto convenuto nella *Consensus* Sepsis-3, **la sepsi** è una *"disfunzione d'organo a rischio-vita conseguente a una risposta de-regolata dell'organismo a un'infezione"* e la sua diagnosi è affidata ad un aumento acuto di due punti del SOFA score rispetto al suo valore di baseline.

Lo **shock settico** è un *"subset della sepsi in cui le alterazioni emodinamiche, cellulari e metaboliche sono di gravità tale da incrementare in misura sostanziale il rischio di morte"* e la sua diagnosi affidata alla presenza di ipotensione refrattaria ad un adeguato reintegro volemico, che richiede l'avvio dell'infusione di farmaci vasopressori per garantire il mantenimento di una PAM $\geq$ 65 mmHg, associata ad un livello di lattati > 2 mmol/l.

Con la nuova definizione, il Sepsis-3 mira a distinguere l'infezione, caratterizzata da un'appropriata risposta infiammatoria dell'ospite, dalla sepsi, in cui la risposta infiammatoria all'infezione è abnorme e lesiva per l'organismo che la produce, si traduce in un danno d'organo e si associa ad una più elevata mortalità ospedaliera.

Il **SOFA** è lo score di riferimento per la misura del danno d'organo nella diagnosi di sepsi e si basa sulla valutazione dello stato funzionale di 6 organi/sistemi, che richiede anche l'ausilio del laboratorio. Poiché la misura delle funzioni d'organo viene quotidianamente eseguita nei reparti di **Terapia Intensiva e in Rianimazione**, il calcolo dello score nei pazienti che in questi setting presentino un'infezione presunta o documentata o che siano ad alto rischio di sepsi può essere immediato e consentire una diagnosi tempestiva.

In **area non critica**, data la tempistica necessaria alla misura dello score, l'approccio al paziente con infezione accertata/sospetta o ad alto rischio di sepsi si avvale di una **fase di *screening*,** volta alla stratificazione del rischio di deterioramento clinico mediante *scores* semplificati di disfunzione d'organo e sistemi di allerta precoce. Tali strumenti si basano su una misura strutturata e ripetibile di alcuni parametri vitali, non necessitano dell'ausilio del laboratorio e consentono di intercettare tempestivamente i pazienti che richiedono un intervento diagnostico-terapeutico immediato. In caso di positività dello *screening*, a questa fase farà seguito la **conferma diagnostica** mediante il SOFA score.

Le principali caratteristiche e i setting ospedalieri di impiego dei **sistemi di *screening* per la sepsi** nella popolazione generale sono di seguito riportati.

- Il **qSOFA,** introdotto dalla *Consensus* Sepsis-3, è di semplice e rapida misura e si basa sulla rapida rilevazione di 2 parametri vitali (pressione arteriosa sistolica, frequenza respiratoria) e dello stato di coscienza. I pazienti con elevata probabilità di esito sfavorevole possono essere rapidamente identificati da un qSOFA ≥ 2. Per la sua bassa sensibilità, il ruolo predittivo di sepsi dello score è stato negli ultimi anni ridimensionato. Pertanto, la sua negatività non deve distogliere gli operatori dal sospetto di sepsi, qualora la clinica lo supporti. Tuttavia, per la sua alta specificità, il qSOFA mantiene ancora la sua valenza come sistema di allarme al **triage** in Pronto Soccorso (all.1).
- Stesso *setting* di impiego è stato identificato per lo **Shock Index**, definito dal rapporto tra la frequenza cardiaca e la pressione arteriosa sistolica ed espressione di ipovolemia (all.1). Lo SI è un utile strumento di rilevazione di una sepsi occulta, soprattutto quando i tradizionali segni clinici sono in apparenza nell'ambito della normalità.
- Più articolato e destinato all'impiego in **Pronto Soccorso e nei reparti di degenza ordinaria** di area sia medica che chirurgica è il **sistema di allerta rapido NEWS**. Il sistema consente la stratificazione della gravità clinica dei pazienti e, sulla base di valori soglia numerici, definisce l'intensità e la rapidità di intervento richieste (all.1). La sua frequenza di rilevazione si basa sul rischio di evolutività e sul giudizio clinico (all.1).

La mancanza di una definizione standardizzata e di criteri diagnostici validati di sepsi per la **popolazione ostetrica** sono tra le principali ragioni chiamate in causa come responsabili del ritardo diagnostico e terapeutico osservato in questo *setting*. Nel 2017, l'OMS ha raccomandato che la **definizione di sepsi e di shock settico materni** ricalchino quelle condivise per la popolazione adulta generale nella *Consensus* Sepsis-3. Tuttavia, ritenendo la misura del danno d'organo mediante il SOFA score non sufficientemente validata in una popolazione in cui i *cut-off* di normalità di alcuni parametri impiegati si discostano da quelli della popolazione generale, l'OMS non ha fornito i criteri diagnostici né di sepsi, né di shock settico.

Società scientifiche del settore hanno proposto che la **diagnosi di sepsi materna** si basi sull'**incremento acuto di un punto** del **SOFA score, modificato** (mSOFA) (all.2) sulla base delle fisiologiche alterazioni della gravidanza, in pazienti con infezione sospetta o documentata. Al fine di intercettare tempestivamente pazienti a rischio di rapida evoluzione, anche in questo setting è prevista una fase preliminare di screening sistematico in tutte le pazienti ospedalizzate mediante il sistema di allerta **MEOWS** (all.1).

I setting ospedalieri e le strategie di impiego dei sistemi di screening e diagnosi di sepsi sono riportati in tabella 1.

Tabella 1. **Strumenti per lo screening e per la diagnosi di sepsi nei diversi setting ospedalieri.**

	PS centrale		Degenza ordinaria Area medica e chirurgica	PS ostetrico e reparto di Ostetricia	UTI e Rianimazione
	**SCREENING DELLA SEPSI**				
**Shock Index**					
**Screening alto rischio***	x				
**Screening infezione****	x				
**qSOFA**					
**Screening alto rischio***	x				
**Screening infezione****	x				
**NEWS**					
**Screening alto rischio***	x		x		
**Screening infezione****	x		x		
**MEOWS**					
**Screening routinario*****				x	
	**DIAGNOSI DI SEPSI**				
**SOFA score**					
**Misura se screening positivo**	x		x		
**Misura routinaria se infezione**					x
**Misura routinaria se alto rischio**					x
**mSOFA score**					
				x	

** Screening alto rischio: lo score viene misurato con sistematicità nelle categorie ad alto rischio di sepsi, indipendentemente dalla presenza o meno di infezione presunta o documentata.*
*** Screening infezione: lo score viene misurato in presenza di infezione sospetta o documentata in categorie di*

pazienti non ad alto rischio.
**** Screening routinario: lo score viene misurato con sistematicità in tutte le pazienti ospedalizzate, sin dall'ingresso in PS (se ricovero in urgenza) o dal ricovero in reparto (se elezione), durante il percorso nascita.*

Il valore aggiunto dei sistemi di allerta rapida nella popolazione non intensivistica, generale e ostetrica, è il loro duplice potenziale:

- di allerta sul rischio di sepsi/shock settico incipiente o conclamato in presenza di infezione documentata o presunta o di un alto rischio di infezione o sepsi (all.3);
- di motivo di ricerca di un'infezione occulta responsabile di una sepsi che passerebbe altrimenti misconosciuta al suo esordio, in caso di riscontro di un danno d'organo non altrimenti giustificabile.

In entrambi i casi, la certezza diagnostica richiederà la misura del SOFA score (all.2) e del mSOFA score.

6.3 Approccio immediato al paziente con sepsi grave/shock sospetti o documentati: il *bundle Sepsis Six* modificato.

Sepsi e shock settico rappresentano condizioni cliniche di emergenza e richiedono un intervento immediato, basato per la **popolazione adulta generale** su misure ben codificate dalla *Surviving Sepsis Campaign* (SSC) e racchiuse nel ***bundle Sepsis Six***.

L'utilità del bundle è di consentire un trattamento tempestivo che segua un flusso operativo definito per ciascun membro del team, in tempi prestabiliti e con obiettivi noti.

Il bundle consta di **3 misure diagnostiche** (misura dei lattati, prelievo per emocolture e monitoraggio della diuresi) e **3 misure terapeutiche** (somministrazione di O_2 in caso di ridotta SpO_2, rianimazione fluidica in caso di ipotensione e somministrazione della terapia antibiotica empirica) (fig.1), da completare **entro la prima ora** dal sospetto clinico (all.4) e garantisce un approccio a dimostrata efficacia su morbilità e mortalità da sepsi. Alle misure suddette, si aggiunge l'avvio della **ricerca del focus infettivo**, la cui eradicazione, se possibile, ha influenza diretta sulla prognosi di questi pazienti.

In accordo alle linee guida della SSC, **entro la terza ora** dall'esordio sarà indispensabile procedere alla rivalutazione del livello di lattati se in precedenza alterato, al monitoraggio della rianimazione fluidica, alla gestione emodinamica avanzata con vasopressori in caso di ipotensione refrattaria, al prosieguo della ricerca e all'avvio del controllo della fonte di infezione.

Nonostante la **popolazione ostetrica** non sia contemplata nelle linee guida della SSC, l'avvio delle misure previste dal bundle è raccomandato anche in questo *setting*, con l'aggiunta della **profilassi anti-tromboembolica** e con opportune precauzioni dettate dalle peculiarità dell'unità materno-fetale.

Il razionale e le modalità di attuazione del bundle nella pratica clinica sono descritti in dettaglio in **allegato 4**.

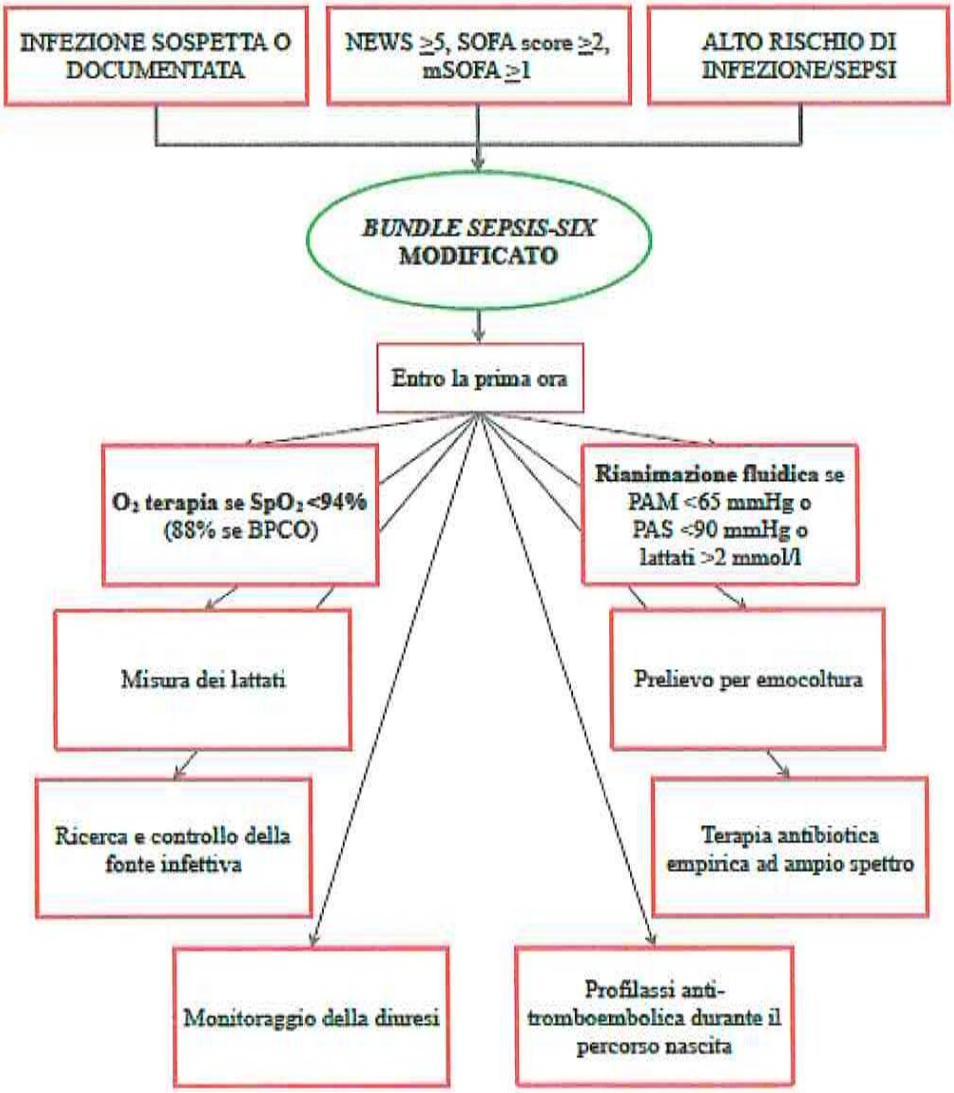

Figura 1. Bundle Sepsis Six modificato.

6.4 Percorso diagnostico-terapeutico-assistenziale dei pazienti con infezione o sepsi presunta/documentata in ospedale.

Il percorso descritto mira ad ottimizzare l'identificazione e la gestione iniziale del paziente settico, eliminando o contenendo i ritardi evitabili.

Tutti i professionisti del settore sono tenuti a conoscere la strategia di screening del rischio evolutivo, i criteri diagnostici di sepsi e di shock settico e le modalità di attuazione del bundle *Sepsis Six* modificato da adottare di fronte ad un paziente con infezione presunta o documentata o ad alto rischio di sepsi o che presenti una disfunzione d'organo non altrimenti giustificabile, e devono essere consapevoli della potenziale rapida evolutività e letalità della sindrome.

La **diagnosi di sepsi** si basa sulla presenza di un'infezione presunta o documentata e di un incremento acuto ≥ 2 punti del SOFA score rispetto al valore di baseline nella popolazione generale e ≥ 1 punto del mSOFA score nella popolazione ostetrica (all. 2);

Sia nella popolazione generale, che nella popolazione ostetrica, la **diagnosi di shock settico** si basa sulla presenza di ipotensione refrattaria ad adeguato reintegro volemico, che richieda l'impiego di farmaci vasopressori per mantenere una PAM ≥ 65 mmHg e si associ a un livello di lattati sierici > 2 mmol/l, in pazienti con infezione presunta o accertata.

Nella gestione del paziente ad alto rischio il medico che ha in carico il paziente potrà avvalersi del **team sepsi** che metterà a disposizione, se necessario, le competenze specialistiche utili alla sua gestione (infettivologo, radiologo, radiologo interventista, chirurgo, anestesista-rianimatore, microbiologo, medico e infermiere della DS esperti in sepsi, referente sepsi del reparto in cui è gestito il paziente), al fine di scegliere il percorso più appropriato per il paziente, tenendo conto dell'evolutività del quadro clinico, dell'eventuale necessità di procedure, del livello più appropriato di monitoraggio e dell'intensità delle cure richieste.

6.4.1 PRONTO SOCCORSO CENTRALE.

Il tempo "zero" nella diagnosi di sepsi in PS corrisponde al momento d'ingresso del paziente in *triage*.

- Al fine di garantire l'identificazione del maggior numero possibile di pazienti a rischio, nei pazienti con infezione presunta o documentata e nei pazienti ad alto rischio di infezione/sepsi (all.3), l'infermiere di triage provvederà alla misura del **qSOFA**, dello **SI** e del **NEWS** (all.1).
- Sulla base della positività o meno dello *screening* e della presenza o meno di fattori di rischio per sepsi o di altri segnali di allarme, il rischio evolutivo verrà stratificato in basso, intermedio o alto e segnerà il percorso successivo del paziente, come riportato in figura 2 e 3.

- Nella gestione del paziente a **rischio evolutivo intermedio-alto**, il medico urgentista e l'infermiere di Area rossa/arancione, seguiranno **il percorso "alta priorità"**, rispettando gli step di applicazione del bundle sepsis six riportati in figura 2 e descritti in dettaglio in allegato 4.
- Sia per la **diagnosi di sede** che, qualora indicato, per il controllo della fonte d'infezione, si raccomanda l'utilizzo in prima istanza della POCUS da parte del medico urgentista e/o del radiologo. In caso di mancata identificazione ecografica è ragionevole procedere con altre indagini strumentali.
- Il **controllo della fonte** di infezione (drenaggio di raccolte fluide o debridment di tessuto solido infetti, rimozione di corpi estranei, procedure chirurgiche definitive, rimozione di cateteri - vescicali, endocavitari o intravascolari - o di dispositivi impiantabili) dovrà realizzarsi **entro 6-12 ore dalla diagnosi** di sepsi/shock settico, **impiegando la tecnica meno invasiva e più efficace, dopo attenta analisi del rapporto rischi/benefici da parte di un team multidisciplinare di esperti di cui il medico urgentista assume la leadership.**

La misura del SOFA score, la stabilità emodinamica e il livello dei lattati condizionano il percorso successivo del paziente (fig. 3):

- In **assenza** di danno d'organo (SOFA < 2) e, dunque, **di sepsi** e in assenza di un livello di lattati sierici > 2 mmol/l o di ipotensione (PAS < 90 mmHg o PAM < 65 mmHg), il medico urgentista:
  - Dispone che il paziente venga posto in **osservazione** e prescrive la tempistica del monitoraggio di segni e sintomi di infezione, del pannello sepsi, del SOFA score e del NEWS.
  - Stabilisce il piano terapeutico più adeguato in base ai riscontri clinici e strumentali.
  - Valuta la necessità di consulenze specialistiche durante l'osservazione clinica.
- In **presenza** di danno d'organo (SOFA score ≥ 2) e, dunque **di sepsi** oppure di **livelli di lattati sierici ≥ 2 mmol/l** e/o di **ipotensione refrattaria** a reintegro volemico adeguato, il paziente prosegue il percorso ad **alta priorità.**

- **In caso di evoluzione clinica rapidamente ingravescente, sepsi con grave compromissione emodinamica o respiratoria (SOFA per singolo organo/apparato ≥ 3), shock settico o di altri elementi clinici di allarme (grave acidosi metabolica, iperlattacidemia, etc) sarà indispensabile contattare il medico rianimatore.** In tal caso, è opportuno che il medico urgentista, in presenza del medico rianimatore, informi il partner o il familiare più prossimo della paziente sull'alto rischio di evoluzione negativa della sindrome.

- **Definizione della sede di cura.** Il trasferimento del paziente dovrebbe avvenire massimo entro le prime 6 ore, compatibilmente con la disponibilità dei posti letto nel reparto di destinazione.
  La valutazione degli scores di gravità può contribuire a stratificare la probabilità di deterioramento del paziente. Un incremento di 3 punti del SOFA score o un NEWS > 6 nelle prime 6 ore si associa ad un incremento significativo della mortalità. Tali scores possono essere di supporto al giudizio clinico per individuare i pazienti da non ricoverare in degenza ordinaria.

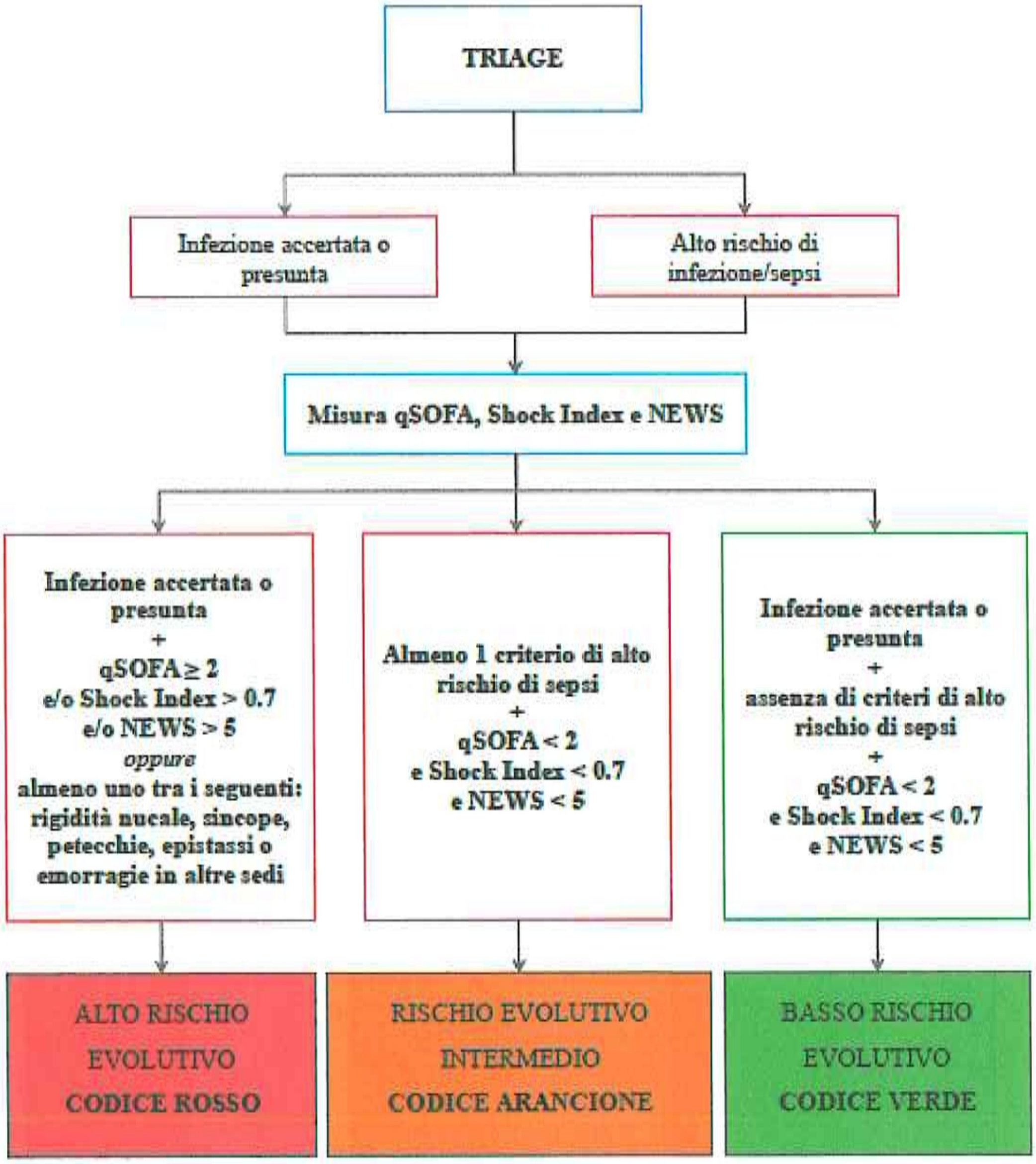

Figura 2. Algoritmo per la stratificazione del rischio evolutivo in Pronto Soccorso centrale

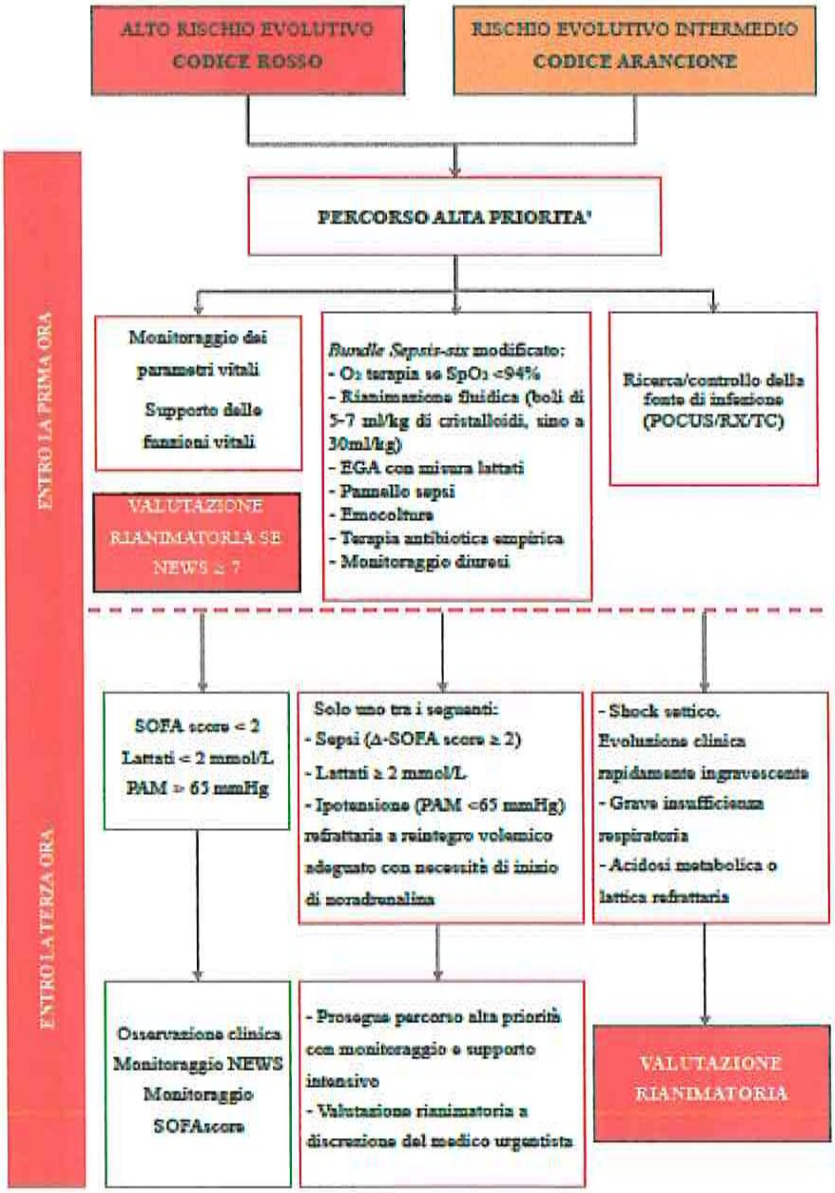

Figura 3. Percorso diagnostico-terapeutico e assistenziale del paziente a rischio evolutivo intermedio-alto in Pronto soccorso.

6.4.2 REPARTI DI AREA MEDICA E CHIRURGICA

- Nei pazienti con infezione presunta o documentata o ad alto rischio di infezione/sepsi (all.3) e nei pazienti che presentino una disfunzione d'organo non altrimenti giustificabile, il medico di guardia dei **reparti di area medica e chirurgica** disporrà il monitoraggio del sistema di allerta **NEWS** (all.1) con frequenza stabilita in base al punteggio dello score (all.1) e registrata sul foglio di terapia.
In caso disfunzione d'organo non giustificabile, disporrà le opportune indagini volte ad escludere un'infezione occulta.
- La misura del NEWS detterà il percorso successivo del paziente (fig.4) e nei pazienti a **rischio evolutivo intermedio-alto**, verrà avviato il percorso "alta priorità" (fig. 5).
  In tal caso, il personale medico e infermieristico avvierà il monitoraggio e il supporto dei parametri vitali (FC, PA, SpO2) e adotterà le misure previste dal bundle Sepsis Six, descritto in dettaglio in allegato 4. Contestualmente, verrà avviata la ricerca della fonte di infezione (se non nota) e si provvederà a quanto necessario alla sua eradicazione se si tratta di un *focus* chirurgico

- In caso di: NEWS $\geq$ 7, ipotensione grave, grave insufficienza respiratoria, acidosi metabolica e/o lattica, verrà richiesto **l'intervento dell'equipe di rianimatori**.

La misura del SOFA score, dei lattati e il valore della PAM, segneranno il percorso successivo del paziente (fig. 5).

- Se il delta SOFA score è < 2, i lattati < 2 mmol/l e la PAM > 65 mmHg, il medico di guardia:

- disporrà che il paziente venga posto in osservazione, avvierà il monitoraggio del NEWS con cadenza definita e scritta sul foglio di terapia, avvierà il monitoraggio almeno quotidiano del pannello sepsi e del SOFA score.
- stabilirà il piano terapeutico più adeguato in base ai riscontri clinici e di laboratorio.
- valuterà la necessità di consulenza infettivologica o di altre consulenze specialistiche.

- In caso di **sepsi** e in assenza di grave disfunzione cardio-circolatoria o respiratoria, proseguirà il percorso alta priorità e la richiesta di **intervento dell'equipe di rianimatori** sarà a discrezione del medico di guardia.

L'intervento dell'equipe di rianimatori sarà indispensabile in caso di **shock settico**, evoluzione clinica rapidamente ingravescente, grave insufficienza respiratoria, acidosi metabolica refrattaria, lattati persistentemente > 2 mmol/l o di qualunque altra condizione che metta il paziente a rischio-vita, si provvederà a richiedere.

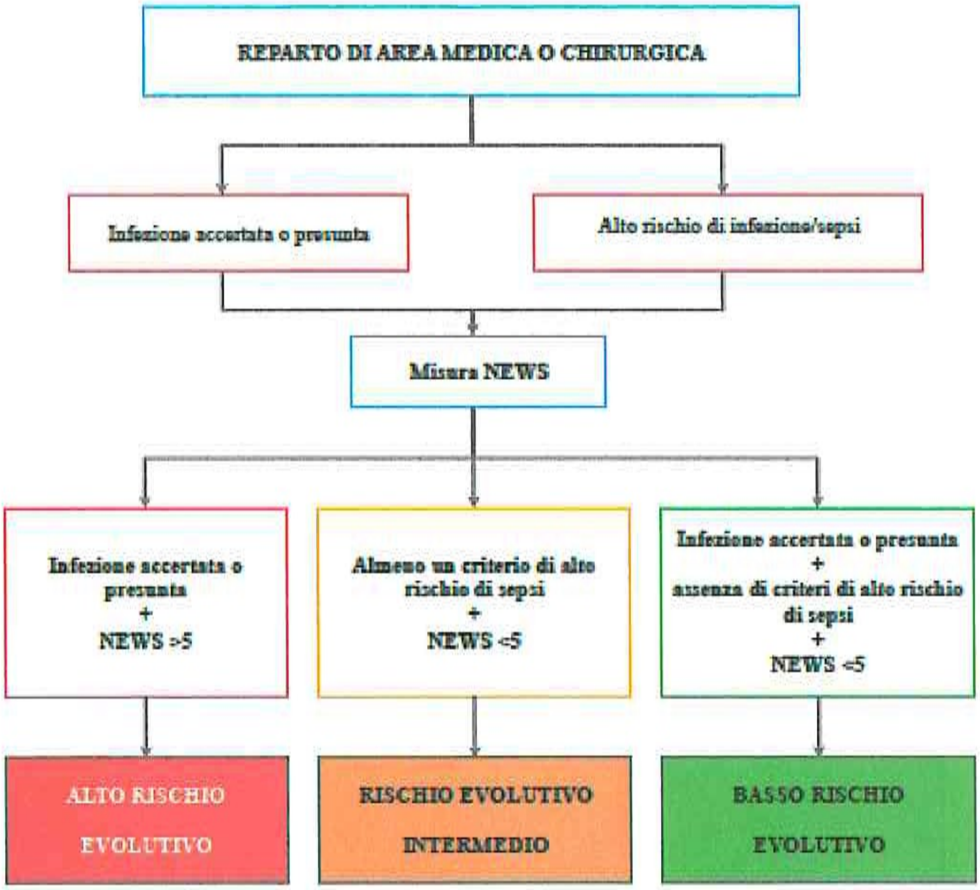

Figura 4. Algoritmo per la stratificazione del rischio evolutivo nei reparti di degenza ordinaria di area medica e chirurgica.

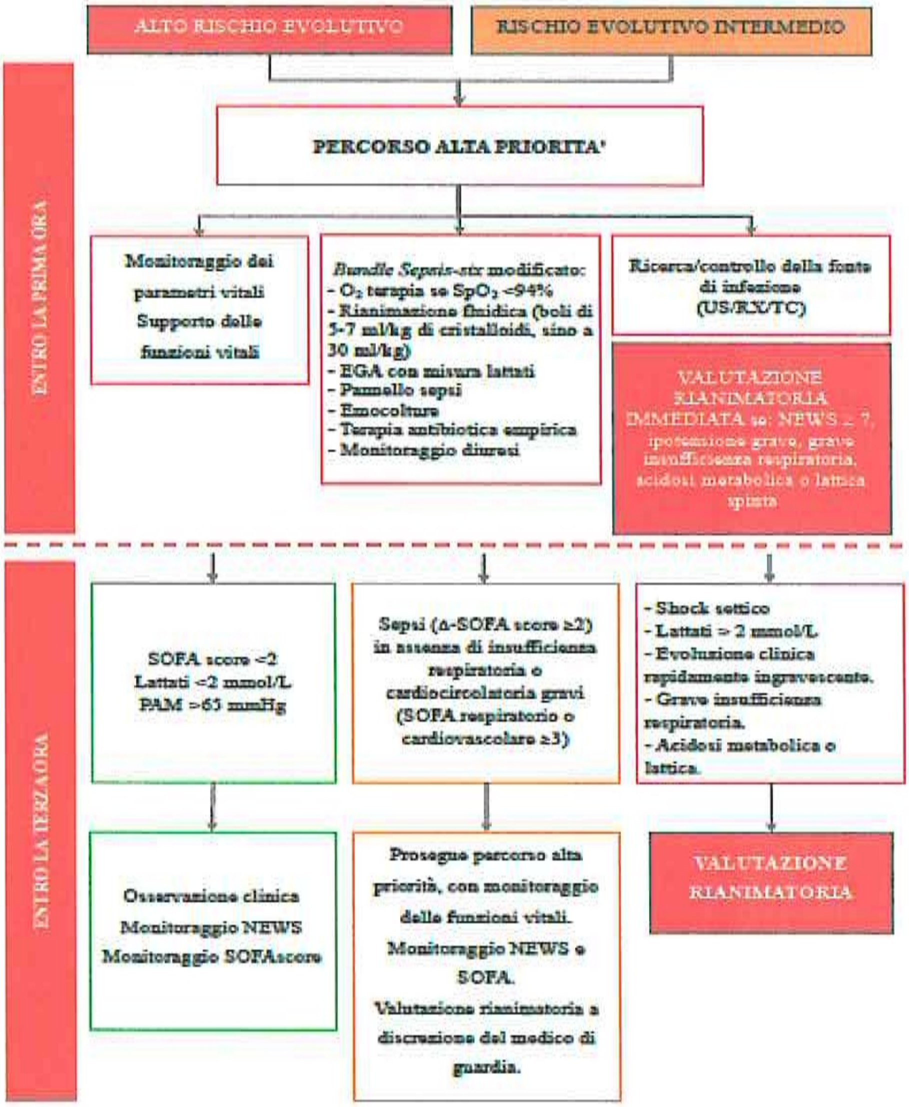

Figura 5. Percorso diagnostico-terapeutico e assistenziale del paziente a rischio evolutivo intermedio-alto nei reparti di area medica e chirurgica.

6.4.3 PRONTO SOCCORSO OSTETRICO E REPARTO DI OSTETRICIA

- **Il personale ostetrico e infermieristico delle UU.OO di degenza, di Sala Parto e di Pronto Soccorso è tenuto al monitoraggio sistematico dei parametri vitali mediante il sistema MEOWS, in tutte le pazienti in gravidanza o entro 42 giorni dal suo esito** che giungano in Pronto Soccorso ostetrico o siano già degenti in reparto, annotando data, ora e operatore (all. 1), con una tempistica prescritta dal medico ginecologo sul foglio di terapia in base al punteggio dello score (all. 1) e ravvicinata in caso di infezione materna documentata o presunta o di alto rischio di infezione/sepsi (tab.3).
- Durante il travaglio, dal momento dell'accesso in sala parto, la rilevazione dei parametri vitali deve essere riportata nel partogramma seguendo il protocollo locale.
- Prima del trasferimento della paziente in reparto dalla sala parto o dalla SO, l'ultima rilevazione dei parametri vitali deve essere riportata sulla scheda MEOWS.
- **Il medico ginecologo** è tenuto a prescrivere sul foglio di terapia la frequenza di rilevazione dei parametri del MEOWS, definita in base alla diagnosi d'ingresso e/o complicanza clinica e al rischio evolutivo definito dall'ultima rilevazione dello score (all 1).
- Tutti i professionisti del settore sono tenuti a conoscere i **criteri diagnostici OMS di sepsi e di shock settico materni** e devono essere consapevoli della potenziale rapida evolutività e letalità della sindrome.

Nella popolazione ostetrica, i possibili **scenari clinici che devono indurre al sospetto di sepsi o di shock settico** sono sostanzialmente tre:

- **Paziente con infezione documentata o presunta** che giunga in Pronto Soccorso Ostetrico o sia già degente in reparto, che presenti un'alterazione di uno o più parametri vitali (≥1 parametro giallo MEOWS).
- Paziente in monitoraggio MEOWS nella quale si riscontri **l'alterazione di uno o più parametri vitali** (≥ 1 parametro giallo) non altrimenti giustificabile.
- Riscontro di ***condizioni fetali non rassicuranti*** non altrimenti giustificabili.

In tali situazioni:

✓ il **medico ginecologo di guardia** è tenuto ad effettuare tempestivamente la valutazione clinica della paziente e del benessere fetale (CTG e/o US);

✓ il **personale infermieristico e/o ostetrico** è tenuto alla rilevazione dei parametri vitali secondo il sistema di allerta MEOWS, qualora non già rilevato.

Il percorso successivo dipenderà dal **livello di rischio evolutivo** definito dal codice colore MEOWS (fig. 6):

1. 1 parametro giallo MEOWS: **basso rischio evolutivo.**

2. 2 parametri gialli o 1 parametro rosso MEOWS: **rischio evolutivo intermedio.**
3. > 2 parametri gialli o > 1 parametro rosso MEOWS: **alto rischio evolutivo.**

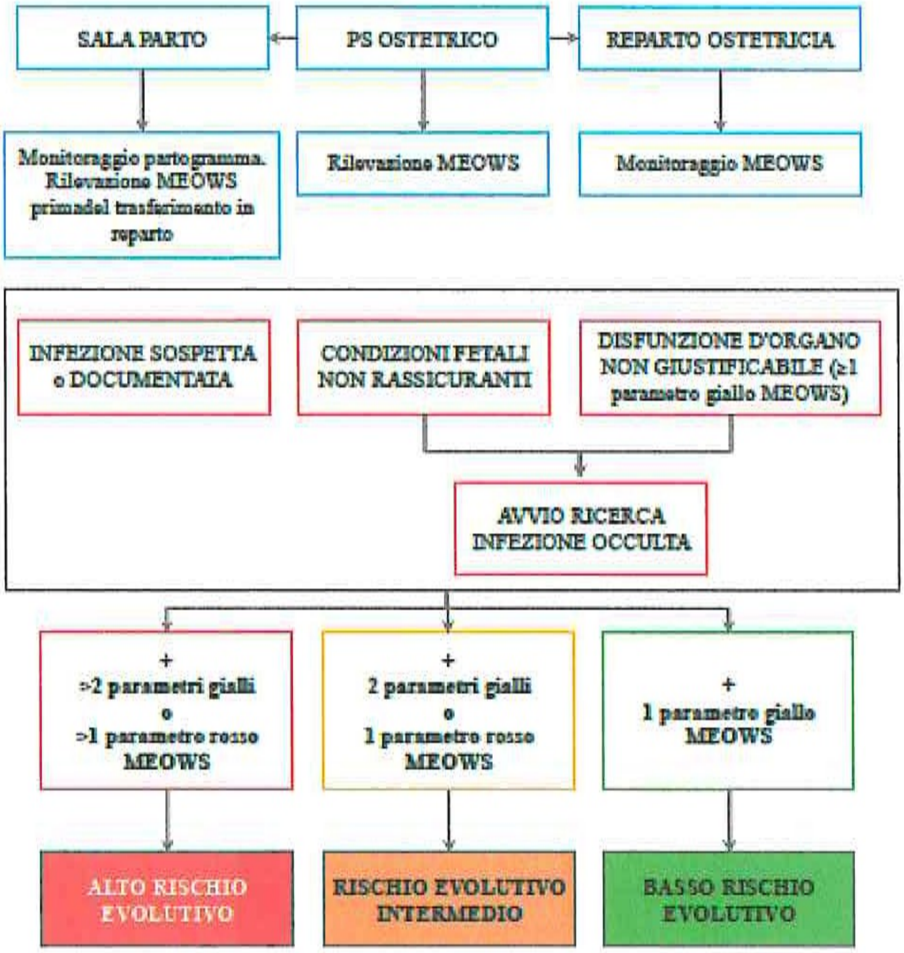

Figura 6. Algoritmo per la stratificazione del rischio evolutivo durante il percorso nascita.

A. Percorso **basso rischio evolutivo.**

Dovrà essere avviato in caso rilevazione di **un parametro giallo** **MEOWS** (fig 7).

Il **medico ginecologo**:

- valutati i parametri vitali materni ed avviata la loro stabilizzazione, dispone la tempistica di rivalutazione del MEOWS;
- dispone l'avvio di emocolture e colture da siti clinicamente sospetti, se non ancora eseguite.
- pondera la necessità di avvio di: pannello sepsi, EGA con lattati, consulenza neonatologica o altre consulenze specialistiche;
- avvia la ricerca della fonte di infezione (se non nota) e provvede a quanto necessario alla sua eradicazione se si tratta di un *focus* chirurgico (evacuazione del prodotto del concepimento, drenaggio di ascessi, espletamento del parto in caso di corionamniosite, isterectomia in caso di necrosi miometriale, risanamento del letto di una ferita chirurgica infetta) o di un dispositivo impiantabile o di un catetere endovasale (CVC, PiCC);
- in caso di fondato sospetto clinico o di certezza d'infezione in corso, avvia subito la terapia antibiotica empirica più idonea alle condizioni della paziente, al sito sospetto o certo d'infezione e all'epidemiologia locale;
- dispone la tempistica del monitoraggio del MEOWS e la registra sul foglio di terapia (all 1).

Il personale ostetrico e/o infermieristico, su disposizione del medico ginecologo:

- ✓ rileva PA, FC, FR, SpO_2 e TC e ricerca sintomi materni ascrivibili ad una corion-amniosite (dolorabilità uterina, liquido amniotico maleodorante, secrezioni cervico-vaginali purulente)
- ✓ incannula una vena periferica di calibro adeguato (18 G);
- ✓ esegue i prelievi per emocolture e per le colture da siti sospetti;
- ✓ esegue i prelievi per pannello sepsi ed EGA con misura dei lattati, se prescritti.
- ✓ prosegue il monitoraggio del MEOWS con la tempistica indicata sul foglio di terapia.

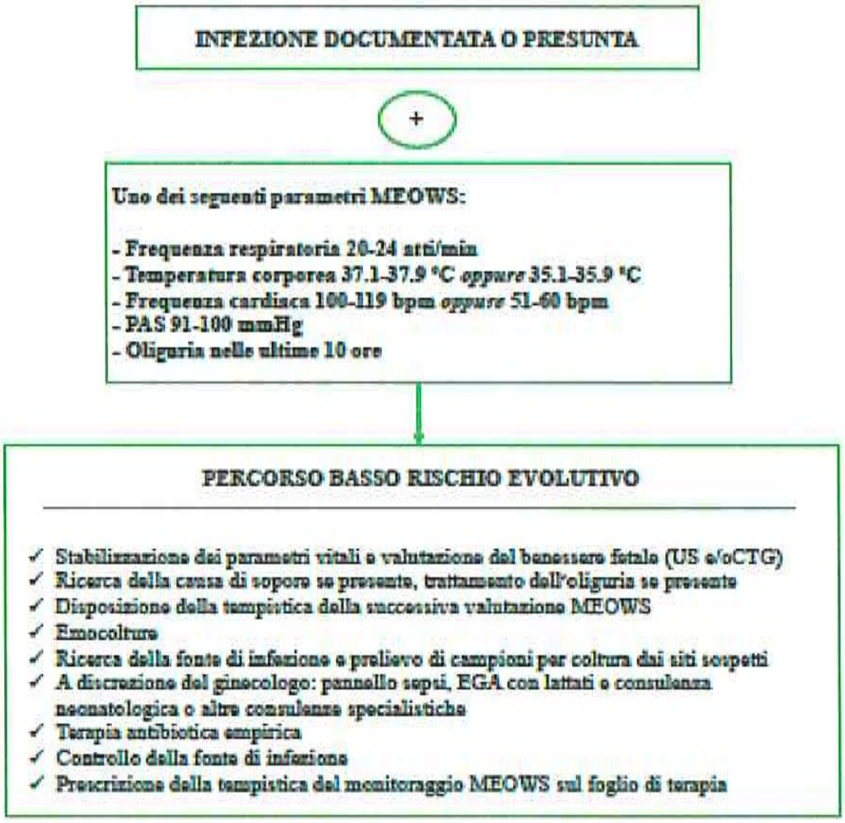

Figura 7. Percorso diagnostico-terapeutico e assistenziale della paziente a basso rischio evolutivo.

B. Percorso rischio evolutivo intermedio.

Viene attivato in caso di rilevazione di almeno 2 parametri gialli o di 1 parametro rosso MEOWS (fig. 8).

Il medico ginecologo, con il supporto dell'anestesista d'urgenza:

- ✓ valutati i parametri vitali materni, provvede al supporto delle funzioni vitali, ove necessario;
- ✓ valuta il benessere fetale (US e/o CTG);
- ✓ avvia contestualmente le misure previste dal *bundle Sepsis six*.

- avvia la ricerca della fonte di infezione (se non nota) e provvede a quanto necessario al suo controllo se si tratta di un *focus* chirurgico (evacuazione del prodotto del concepimento, drenaggio di ascessi, espletamento del parto in caso di corion-amniosite, isterectomia in caso di necrosi miometriale, risanamento del letto di una ferita chirurgica infetta) o di un dispositivo impiantabile o di un catetere endovasale (CVC, PiCC);
- avvia la profilassi anti-tromboembolica secondo il protocollo locale o mediante consulenza al centro emostasi e trombosi;
- dispone la tempistica del monitoraggio dei parametri vitali con sistema MEOWS sul foglio di terapia;
- dispone la tempistica del monitoraggio fetale;
- richiede la consulenza del neonatologo;
- pondera la necessità di consulenza rianimatoria e/o infettivologica.
- pondera la necessità di espletamento del parto, tenendo presente che è indispensabile stabilizzare prima le condizioni materne, tranne in caso di infezione endouterina e corion-amniosite, in cui è previsto il rapido espletamento del parto e, stabilizzate le condizioni materne, la rimozione del materiale ritenuto in utero.

Il personale infermieristico e/o ostetrico, su disposizione del medico ginecologo:

- ✓ rileva PA, FC, FR, SpO2 e TC e ricerca sintomi materni ascrivibili ad una corion-amniosite (dolorabilità uterina, liquido amniotico maleodorante, secrezioni cervico-vaginali purulente);
- ✓ in caso di SpO2 < 94% avvia la somministrazione di ossigeno in maschera;
- ✓ incannula due vene periferiche (16 G, 18 G) e, in caso di ipotensione, avvia la rianimazione fluidica;
- ✓ posiziona il catetere vescicale con urinometro;
- ✓ se la gestazione ha superato la 20^ settimana, posiziona la paziente in decubito laterale sinistro per eliminare una potenziale concausa di ipotensione;

✓ esegue i prelievi per emocoltura e per le colture da siti sospetti e avvia la somministrazione della terapia antibiotica;
✓ esegue i prelievi per pannello sepsi ed EGA con misura dei lattati;
✓ avvia il monitoraggio in continuo dei parametri vitali e della diuresi orari;
✓ prosegue il monitoraggio del MEOWS con la tempistica indicata sul foglio di terapia.

La misura del mSOFA score, la stabilità emodinamica e il livello dei lattati condizionano il percorso successivo della paziente (fig. 8):

o In **assenza** di danno d'organo (mSOFA < 1) e, dunque, **di sepsi** e in assenza di un livello di lattati sierici > 2 mmol/l o di ipotensione (PAS < 90 mmHg o PAM < 65 mmHg), il medico ginecologo:
✓ dispone che la paziente venga posta in osservazione ed avvia il monitoraggio di segni e sintomi di infezione, del pannello sepsi e del MEOWS;
✓ stabilisce il piano terapeutico più adeguato in base ai riscontri clinici e strumentali;
✓ valuta la necessità di consulenze specialistiche durante l'osservazione clinica.

o In **presenza** di danno d'organo (**mSOFA score ≥ 1**) e, dunque, **di sepsi** oppure in presenza di **livelli di lattati sierici ≥ 2 mmol/l** o di **ipotensione** (PAS < 90 mmHg o PAM < 65 mmHg) **refrattaria** a reintegro volemico adeguato, il medico ginecologo prosegue come descritto nel percorso **alto rischio.**

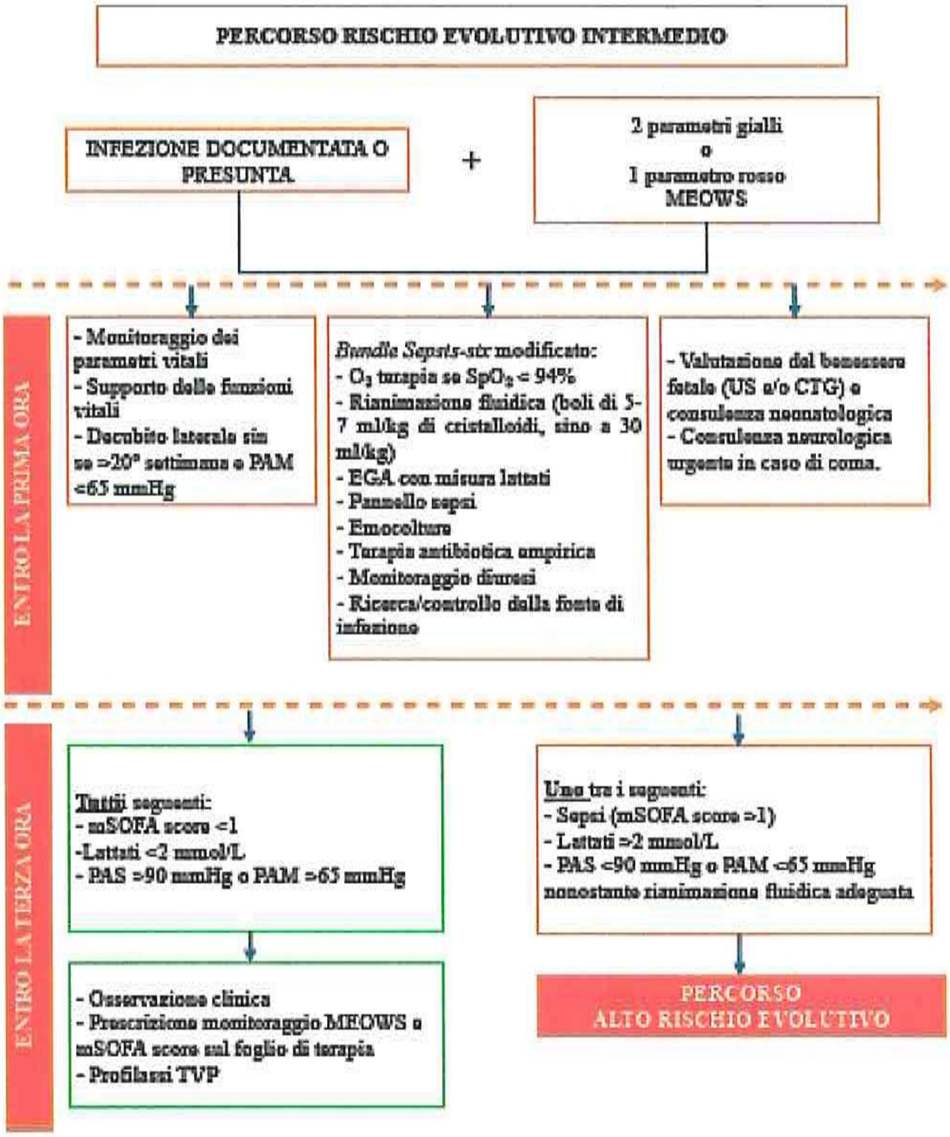

Figura 8. Percorso diagnostico-terapeutico e assistenziale della paziente a rischio evolutivo intermedio.

C. Percorso alto rischio evolutivo.

La condizione di **alto rischio** è definita dalla presenza di un'**infezione presunta o certa, associata a una delle seguenti condizioni:**

- Rilevazione di ≥ 2 parametri gialli e/o di > 1 parametro rosso MEOWS;
- Sepsi (mSOFA score ≥1);
- Livelli di lattati sierici > 2 mmol/l.
- Ipotensione refrattaria ad adeguato reintegro volemico.
- Shock settico

Tale condizione **richiede la valutazione congiunta immediata da parte del medico ginecologo, dell'anestesista-rianimatore, dell'infettivologo di guardia e del neonatologo** e **l'attivazione del team sepsi (fig. 9).**

- In attesa dell'arrivo dei consulenti, il **medico ginecologo** avvia il **percorso "alto rischio"** che prevede:
  - ✓ avvio di quanto previsto nel **percorso rischio intermedio.**
  - ✓ **in attesa dell'arrivo dei rianimatori, richiesta d'intervento immediato dell'anestesista d'urgenza in caso di shock settico, grave desaturazione (SpO_2 < 94% in ossigenoterapia ad alti flussi) o dispnea marcata o grave acidosi metabolica o lattica all'EGA.**
- Al suo arrivo, **il consulente infettivologo** verificherà l'appropriatezza della terapia antibiotica avviata e indicherà eventuali approfondimenti diagnostici utili alla definizione di sede e eziologia dell'infezione con il supporto, se necessario, del team sepsi.
- **Il medico rianimatore** provvederà alla stabilizzazione delle funzioni vitali e alla definizione dell'intensità di cure richieste sulla base della misura del danno d'organo e dell'evolutività del quadro clinico, con il supporto, se necessario, del team sepsi.
- **Il team sepsi** analizzerà il caso in tempi brevi e con un approccio multidisciplinare, mettendo a disposizione, se necessario, le competenze specialistiche utili alla sua gestione (infettivologo, radiologo, radiologo interventista, chirurgo, anestesista-rianimatore, microbiologo, medico e infermiere della DS esperti in sepsi) e supportando il medico ginecologo e i consulenti nella scelta del percorso più appropriato per la paziente, tenendo conto dell'evolutività del quadro clinico, dell'eventuale necessità di procedure, del livello più appropriato di monitoraggio e dell'intensità delle cure richieste.
- Il **medico ginecologo e i consulenti infettivologo e rianimatore, con il supporto del team sepsi**, stabiliranno inoltre quanto necessario al controllo del focus d'infezione se si tratta di focus eradicabile chirurgicamente (evacuazione del prodotto del concepimento, drenaggio di ascessi, espletamento del parto in caso di corion-amniosite, isterectomia in caso di necrosi miometriale, risanamento del letto di una ferita chirurgica infetta) o di un dispositivo impiantabile o di un catetere endovasale (CVC, PiCC) (all.4).

o Il personale infermieristico e/o ostetrico, su disposizione del medico ginecologo, espleterà le attività previste dal percorso "rischio intermedio" e collaborerà all'espletamento delle indagini diagnostiche e alla stabilizzazione e al monitoraggio delle funzioni vitali della paziente.

In caso di **stabilizzazione delle condizioni cliniche** e prosieguo delle cure presso la U.O di ostetricia e ginecologia, il **medico ginecologo**:

- avvierà la profilassi anti-tromboembolica secondo il protocollo locale o mediante consulenza del centro emostasi e trombosi;
- disporrà la tempistica del monitoraggio del MEOWS e del mSOFA score sul foglio di terapia;
- disporrà la tempistica del monitoraggio fetale.

In caso di evoluzione clinica rapidamente ingravescente, sepsi con grave insufficienza di uno o più organi/apparati, shock settico, necessità di ventilazione meccanica o di altri elementi clinici di allarme per il medico rianimatore (grave acidosi metabolica, iperlattacidemia, etc), si provvederà al trasferimento della paziente in Terapia Intensiva.

In tal caso, è opportuno che il medico ginecologo, in presenza del medico rianimatore e dell'infettivologo, informi il partner o il familiare più prossimo della paziente sull'alto rischio di evoluzione negativa della sindrome.

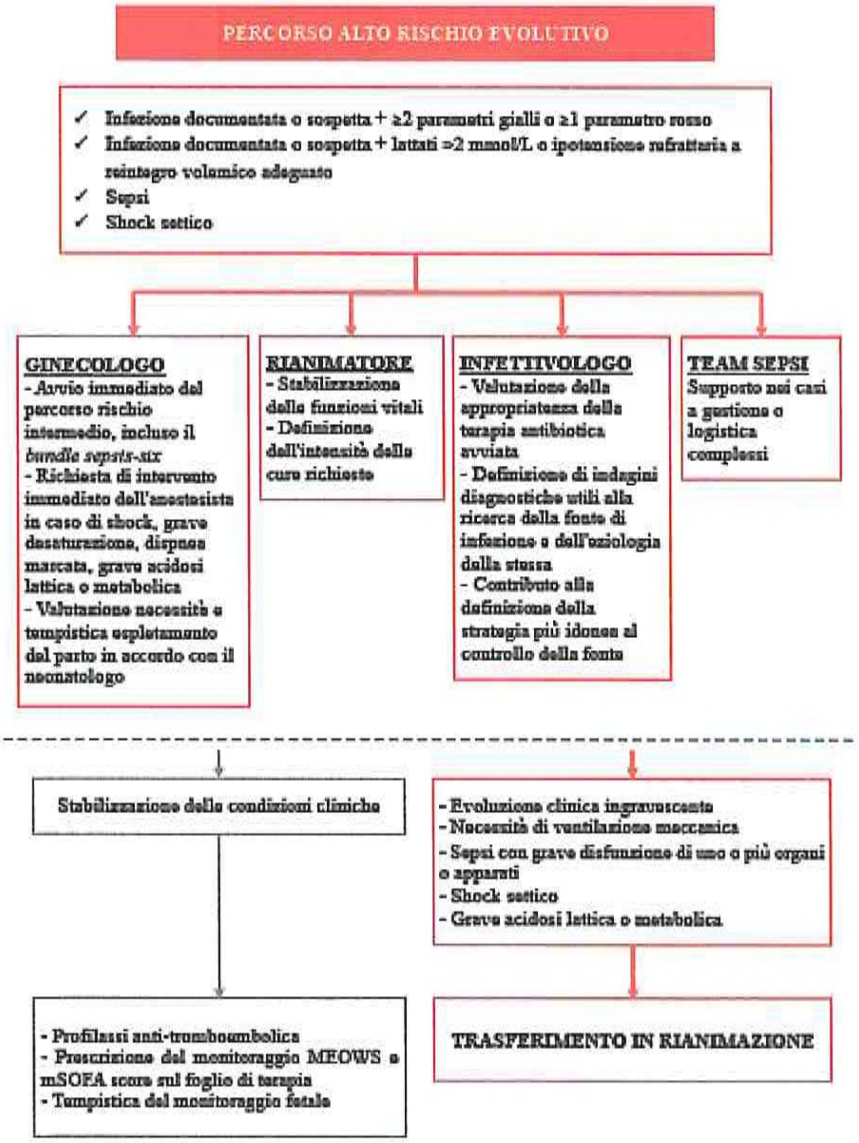

Figura 9. Percorso diagnostico-terapeutico e assistenziale della paziente ad alto rischio evolutivo.

6.4.4 UNITA' DI TERAPIA INTENSIVA SPECIALISTICA E REPARTI DI RIANIMAZIONE.

- Nelle **Unità di Terapia Intensiva** e nei **reparti di Rianimazione** è previsto che si provveda al monitoraggio quotidiano del **SOFA** score (all. 2):
  - in tutti i pazienti con infezione presunta o certa,
  - nei pazienti ad alto rischio di infezione/sepsi (all. 3)
  - nei pazienti che presentino una disfunzione d'organo non altrimenti giustificabile. In quest'ultima categoria di pazienti, si avvieranno le indagini necessarie alla ricerca di un'infezione occulta che possa sottendere la disfunzione d'organo riscontrata.

- In presenza di un delta SOFA score ≥ 2 e di un'infezione documentata o presunta, si porrà diagnosi di sepsi e si procederà all'avvio del bundle Sepsis six (all.4).

- In presenza di shock settico, superata la fase di rianimazione fluidica, sarà indispensabile eseguire la fase di **ottimizzazione emodinamica** (all.4) sotto la guida di un monitoraggio emodinamico invasivo, della misura dei lattati e della misura della ScvO2 (fig. 10).

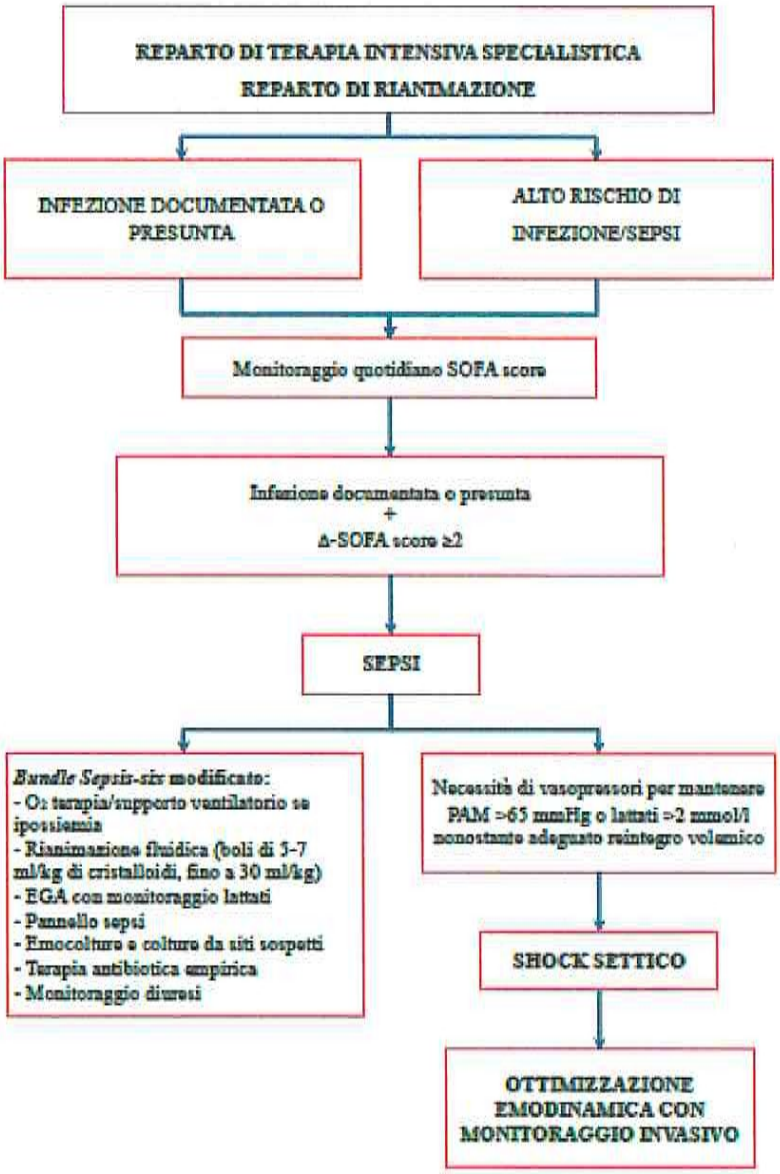

Figura 10. Primo approccio al paziente con sepsi o shock settico nelle TI specialistiche e in rianimazione.

7. VERIFICA DEI PROCESSI E DELLE PRESTAZIONI

I processi devono essere verificati a cadenza annuale dai responsabili delle UU.OO. che hanno avuto un ruolo nel processo e dalla UQA per quanto concerne i documenti che interessano tutta l'AOU. I documenti dovranno essere sottoposti a revisione almeno ogni 12 mesi e comunque ogni qualvolta una modifica/integrazione organizzativa, gestionale o normativa lo richieda. Ogni documento revisionato abroga automaticamente la versione precedente del documento stesso.

Farà parte della modalità operativa l'individuazione di indicatori di processo e di risultato.

Indicatore di Processo	Valore Limite	Valore Atteso
N° test di screening compilati/n° totale di pazienti ricoverati in 1 anno nei reparti di ostetricia e in Pronto soccorso ostetrico	60%	100%
N° pazienti accolti con sospetta sepsi in triage/N° pazienti ricoverati con diagnosi di sepsi o shock settico	50%	80%
N° infermieri-ostetriche-medici formati in 1 anno sul percorso Sepsi /n° totale di infermieri-ostetriche-medici	50%	100%

Indicatore di Esito	Valore Limite	Valore Atteso
N° pazienti con diagnosi SDO di shock settico deceduti/n° totale di pazienti con diagnosi SDO di shock settico.	35%	20%

8. AZIONI DI MIGLIORAMENTO

- A tutto il personale coinvolto e al team sepsi verrà illustrata la presente procedura in un incontro preliminare, che prevede anche momenti di addestramento sulla corretta compilazione e tempistica di rilevazione dei sistemi di screening e sul corretto approccio diagnostico-terapeutico e assistenziale nei rispettivi setting.

- È previsto lo svolgimento periodico d'incontri informativi e formativi rivolti a tutto il personale medico e di comparto dell'ospedale e al team sepsi, volti ad ottimizzare la tempestività e appropriatezza degli interventi previsti dal PDTA.
- Si provvederà poi, con periodicità, alla verifica o audit dell'aderenza a quanto previsto dal PDTA con restituzione dei dati. Tale approccio rappresenta da un lato un importante strumento di verifica, dall'altro un efficace momento formativo per il personale.

Sarà cura del team sepsi:

- definire percorsi diagnostico-terapeutici di controllo/eradicazione per le principali fonti di infezione, al fine di ottimizzare la tempistica e le modalità di gestione del paziente, tenendo conto delle risorse logistico-organizzative.
- definire indicazioni e percorsi di utilizzo della diagnostica molecolare rapida.
- definire un approccio multi-markers di supporto alla diagnosi di infezione e al monitoraggio della risposta alla terapia.
- promuovere l'aggiornamento della terapia antibiotica empirica sulla base dei report sull'epidemiologia locale forniti dal laboratorio di microbiologia.

9.IDENTIFICAZIONE, EMISSIONE E ARCHIVIAZIONE DEI DOCUMENTI

9.1 Identificazione ed emissione

L'identificazione e l'emissione della procedura aziendale saranno a carico del UQA.

9.2 Archiviazione

L'archiviazione e la conservazione del Processo sarà a cura della Segreteria degli Atti Amministrativi.

10. REDAZIONE E REVISIONE DEL DOCUMENTO E GRUPPO DI LAVORO

REDAZIONE E REVISIONE DEL DOCUMENTO	
Dott.ssa L. Dalfino	U.O.C Anestesia e Rianimazione Universitaria II "A. Brienza"
Dott. Piero Pozzessere	U.O.C Pronto Soccorso
Prof.ssa A. Vimercati	U.O.C Ginecologia e Ostetricia
Prof. S. Grasso	U.O.C Anestesia e Rianimazione Universitaria II "A. Brienza"
Prof. A. Dell'Erba	U.O Gestione Rischio Clinico e Sicurezza dei Pazienti
GRUPPO DI LAVORO	
Dott. G. Calabrese	Direzione Sanitaria
Dott. M. Marucci	U.O.C Anestesia e Rianimazione Universitaria II "A. Brienza"
Dott.ssa F. Zotti	U.O Gestione Rischio Clinico e Sicurezza dei Pazienti
Dott.ssa F. Carpagnano	Scuola di specializzazione in Igiene e Medicina Preventiva – Direzione Sanitaria
Dott.ssa M. Stufano	Scuola di specializzazione in Anestesia, Rianimazione, Terapia Intensiva e Terapia del dolore
Dott.ssa V. Rafaschieri	Direzione Sanitaria

Processo Operativo

PDTA per l'Identificazione Precoce e la Gestione Tempestiva della Sepsi nell'adulto

11. ALLEGATI

ALLEGATO 1.

STRUMENTI PER LO SCREENING DELLA SEPSI.

Quick SOFA (qSOFA).

- Setting di impiego: **triage** in Pronto Soccorso Centrale

Parametro	Punteggio
Pressione Arteriosa Sistolica < 100 mm Hg	1
Stato di coscienza AVPU **score in** Verbal o peggiore	1
Frequenza respiratoria ≥ 22 atti/min	1

Il qSOFA è positivo quando il punteggio dello score è ≥ 2

BOX 1. LA RILEVAZIONE DEL qSOFA NELLA PRATICA CLINICA.

1. **Rilevazione della Pressione Arteriosa.** Può essere effettuata con sfigmomanometri elettronici con bracciale adeguato alle dimensioni del braccio del paziente. In caso di misura ritenuta poco attendibile o della rilevazione di valori gravemente patologici, è opportuno procedere alla misura con sfigmomanometro manuale. **Una PAS < 100 mmHg è segno frequente di sepsi e va immediatamente segnalata al medico di guardia.**
2. **Rilevazione della frequenza respiratoria (FR).** La tachipnea è un segno precoce di sepsi e richiede un'approfondita valutazione. La FR si misura in atti al minuto, contando gli atti respiratori (escursioni toraciche) per 30 secondi e moltiplicandoli per due se il respiro è regolare, altrimenti il conteggio va proseguito per 60 secondi. È bene evitare che il paziente si accorga della misurazione e cambi il suo modo di respirare. **Un valore di FR > 22 atti/min va segnalato al medico di guardia.**
3. **Valutazione dello stato di coscienza.** Viene eseguita mediante la scala AVPU:

- **A** – il paziente è vigile, orientato nel tempo e nello spazio (Alert).
- **V** – il paziente è soporoso e risponde solo al richiamo verbale (Verbal).
- **P** – il paziente è soporoso e risponde solo a stimoli dolorosi con movimenti volontari o non volontari (Pain).
- **U** – il paziente non è responsivo ad alcuno stimolo (Unresponsive).

Qualunque risposta diversa da quella definita in A è segno di allarme e va immediatamente segnalata al medico di guardia.

SHOCK INDEX (SI)

- Setting di impiego: **triage** in Pronto Soccorso Centrale

SI = FREQUENZA CARDIACA/PRESSIONE ARTERIOSA SISTOLICA

In condizioni normali, il valore dello SI è 0.5-0.7.
Uno SI > 1 è un indicatore di rapida evoluzione negativa in assenza di interventi.

NEW EARLY WARNING SYSTEM (NEWS)

- Setting di impiego: **Pronto Soccorso Centrale, reparti di Area Medica e Chirurgica**.

Il NEWS si basa sulla misurazione di 7 parametri fisiologici.

National Early Warning System (NEWS)

	PUNTEGGIO						
Parametri Fisiologici	3	2	1	0	1	2	3
FR (atti/min)	≤ 8		9-11	12-20		21-24	≥ 25
SpO2	≤ 91%	92-93%	94-95%	≥ 96%			
O2 terapia		Si		No			
PAS (mmHg)	≤ 90	91-100	101-110	111-219			>220
FC (battiti/min)	≤ 40		41-50	51-90	91-110	111-130	≥ 131
Coscienza				Vigile			CVPU
TC (°C)	≤ 35		35.1-36	36.1-38	38.1-39	≥ 39.1	

Mediante l'identificazione di due valori soglia, stratifica i livelli di rischio e l'intensità dell'intervento richiesto in urgente (valore soglia dello score 5) e in emergenza (valore soglia dello score 7).

BOX 2. LA RILEVAZIONE DEL NEWS NELLA PRATICA CLINICA.

- **Rilevazione della frequenza respiratoria.** Vedi qSOFA.
- Misurazione della **Saturazione periferica di Ossigeno (SpO_2).** Valori normali di SpO_2 sono compresi tra 96% e 100%. Quando la circolazione periferica è compromessa (estremità fredde), la misura della SpO_2 può non essere attendibile o il parametro può non essere rilevabile. Una alternativa può essere porre il sensore sul lobo auricolare, anziché a livello digitale.
- **Rilevazione della pressione arteriosa.** Vedi qSOFA
- **Rilevazione della frequenza cardiaca.** La sede più accessibile di rilevazione è il polso radiale. In caso di ipotensione, in cui il polso radiale può essere assente, la misura va effettuata a livello brachiale o femorale o carotideo. La misura va eseguita palpando l'arteria con dito indice e medio, contando i battiti per 30 secondi e moltiplicando per due se il battito è regolare o proseguendo per 60 secondi se il battito è irregolare. Alcuni strumenti elettronici (saturimetro, sfigmomanometro, monitor ECG) forniscono una misura attendibile della FC. I valori normali di FC sono compresi tra 50 e 90 battiti/minuto.
- **Valutazione del livello di coscienza.** Viene eseguita mediante la scala AVPU: Vedi qSOFA
- **Rilevazione della temperatura corporea.** La sede di rilevazione più adeguata è quella timpanica. Valori normali di TC sono compresi tra 36°C e 37.4 °C. Il riscontro di ipotermia (TC < 36°C) non è infrequente, soprattutto nei pazienti in età avanzata o defedati e non va ignorato poiché, come l'iperpiressia, può essere indicativa di infezione o sepsi.

Tabella 1. **Stratificazione del rischio evolutivo in base al punteggio NEWS**

NEWS Score	Rischio	Risposta
0-4	Basso	Risposta del reparto
5-6	Intermedio	Soglia per la risposta urgente
≥ 7 o presenza di un solo parametro con punteggio 3	Alto	Risposta in emergenza o urgenza

Tabella 2. **Frequenza di rilevazione del NEWS**

NEW Score	Frequenza di monitoraggio	Risposta clinica
**0**	Minimo ogni 12 ore	• Monitoraggio routinario
**1-4**	Ogni 4-6 ore	• Condividere il segnale di allerta durante le consegne infermieristiche.<br>• In caso di variazione comunica al medico

**3 per un singolo parametro**	Minimo ogni ora	• L'infermiere informa il medico che deciderà la terapia
**>5 soglia per risposta urgente**	Minimo ogni ora	• L'infermiere richiede al medico una valutazione urgente<br>• Su disposizione del medico fornisce le cure necessarie
**>7 soglia per risposta in emergenza**	Monitoraggio in continuo dei parametri vitali	• L'infermiere richiede al medico una valutazione urgente<br>• Su disposizione del medico e del sepsi team fornisce le cure necessarie

MODIFIED EARLY OBSTETRIC WARNING SYSTEM

- Setting di impiego: **Percorso nascita**

Nel sistema MEOWS adottato in questo PDTA ciascun parametro è graduato in livelli, a ciascuno dei quali è attribuito un codice colore che consente di cogliere immediatamente l'allarme di deterioramento. La somma dei codici colore fornisce la misura dello scostamento dalla fisiologia normale e il tipo di risposta da attuare.

BOX 1. LA RILEVAZIONE DEL MEOWS NELLA PRATICA CLINICA.

La rilevazione di base dello score prevede la misura di:

- ✓ **Frequenza respiratoria**
- ✓ **Temperatura corporea**
- ✓ **Frequenza cardiaca**
- ✓ **Pressione arteriosa**
- ✓ **Livello di coscienza**

Parametri aggiuntivi, da rilevare solo in determinate condizioni sono:

- ✓ **Saturazione periferica di Ossigeno (SpO_2)**
- ✓ **Diuresi**

- **Rilevazione della frequenza respiratoria.** La tachipnea (FR > 19 atti/min) è un segno precoce di sepsi e richiede un'approfondita valutazione. La frequenza respiratoria (FR) si misura in atti al minuto, contando gli atti respiratori (escursioni toraciche) per 30 secondi e moltiplicandoli per due se il respiro è regolare o proseguendo il conteggio per 60 secondi in caso di respiro irregolare. E' bene evitare che la paziente si accorga della misurazione e cambi il suo modo di respirare. Il valore normale della FR durante la gravidanza e il puerperio è di 11-19 atti/min.
- In presenza di tachipnea (FR ≥ 20 atti/min), è indispensabile la misurazione della **Saturazione periferica di Ossigeno (SpO_2)**, parametro aggiuntivo del MEOWS. Quando la

circolazione periferica è compromessa (estremità fredde), la misura della SpO2 può non essere attendibile o il parametro può non essere rilevabile. Una alternativa può essere porre il sensore sul lobo auricolare, anziché a livello digitale. Valori normali di SpO2 sono compresi tra 96% e 100%.

- **Rilevazione della temperatura corporea**. La sede di rilevazione più adeguata è quella timpanica. Valori normali di TC sono compresi tra 36°C e 37.4 °C. Il riscontro di ipotermia (TC < 36°C) non va ignorato poiché, come l'iperpiressia, può essere indicativo di infezione o sepsi.
- **Rilevazione della frequenza cardiaca**. La sede più accessibile di rilevazione è il polso radiale. In caso di ipotensione, in cui il polso radiale può essere assente, la misura va effettuata a livello brachiale o femorale o carotideo. La misura va eseguita palpando l'arteria con dito indice e medio, contando i battiti per 30 secondi e moltiplicando per due se il battito è regolare o per 60 secondi se il battito è irregolare. Alcuni strumenti elettronici (saturimetro, sfigmomanometro, monitor ECG) forniscono una misura attendibile della FC. I valori normali di FC durante la gravidanza e il puerperio sono compresi tra 60 e 99 battiti/minuto.
- **Rilevazione della pressione arteriosa**. Può essere effettuata con sfigmomanometri elettronici con bracciale adeguato alle dimensioni del braccio della paziente. In caso di misura ritenuta poco attendibile o della rilevazione di valori gravemente patologici, è opportuno procedere alla misura con sfigmomanometro manuale. I valori normali di PAS sono compresi tra 100 e 139 mmHg, i valori normali di PAD sono compresi tra 50 e 89 mmHg. L'ipotensione arteriosa (PAS < 90 mmHg) è segno di deterioramento avanzato delle condizioni materne e va immediatamente segnalata al medico di guardia.
- **Valutazione del livello di coscienza**. Viene eseguita mediante la scala AVPU:

A – la paziente è vigile, orientata verso persone, tempo, luogo ed evento (Alert).

V – la paziente è soporosa e risponde al richiamo verbale (Verbal).

P – la paziente è soporosa e risponde solo a stimoli dolorosi con movimenti volontari o non volontari (Pain).

U – la paziente non è responsiva ad alcuno stimolo (Unresponsive).

- **Monitoraggio della diuresi.** Va avviato in caso di oligo-anuria (diuresi oraria < 0.5 ml/ora), riferita dalla paziente o riscontrata oggettivamente in pazienti portatrici di catetere vescicale, che è sempre un segnale di allerta e va immediatamente segnalato al medico di guardia.

Tabella 3. **Frequenza di rilevazione del MEOWS.**

CONDIZIONE CLINICA	FREQUENZA MINIMA DI RILEVAZIONE
**Gravidanza a basso rischio e non complicata (paziente ricoverata).**	• Al ricovero<br>• Ogni 24 ore.<br>• Frequenza incrementabile su indicazione clinica.

**Post-partum: paziente a basso rischio con gravidanza e parto non complicati.**	• Subito dopo il parto.<br>• Ogni 24 ore sino alla dimissione, salvo diversa indicazione clinica.
**Dopo taglio cesareo o dopo chirurgia durante la gravidanza e il periodo post-natale.**	• Ogni 15 minuti in sala risveglio.<br>• Ogni 30 minuti per 2 ore nel post-partum.<br>• Ogni 4-8 ore per le successive 48 ore.<br>• Ogni 24 ore sino alla dimissione.
**Infezione materna sospetta o documentata o alto rischio di infezione/sepsi o condizioni fetali non rassicuranti**	• Ogni 4 ore.<br>• Frequenza incrementabile su indicazione clinica.

SISTEMA DI ALLERTA PRECOCE IN OSTETRICIA (MEOWS – Modified Early Warning Obstetric System)									
CONTATTARE IL MEDICO SE ≥ 1 TRIGGER ROSSO O ≥ 2 TRIGGER GIALLI									
Nome		Cognome					Data di ricovero		
ANNO	DATA								
	ORA								
FREQUENZA RESPIRATORIA (atti/min)	≥ 25								≥ 25
	≥ 20								≥ 20
	11-19								11-19
	≤ 10								≤ 10
SpO_2 (%)	96-100								96-100
	≤ 95								≤ 95
TEMPERATURA CORPOREA (°C)	≥ 38								≥ 38
	37.5-37.9								37.5-37.9
	36-37.4								36-37.4
	35.1-35.9								35.1-35.9
	≤ 35								≤ 35
FREQUENZA CARDIACA (battiti/min)	≥ 120								≥ 120
	100-119								100-119
	61-99								61-99
	60-51								60-51
	≤ 50								≤ 50
PA SISTOLICA (mmHg)	≥ 160								≥ 160
	140-159								140-159
	101-139								101-139
	91-100								91-100
	≤ 90								≤ 90
PA DIASTOLICA (mmHg)	≥ 100								≥ 100
	90-99								90-99
	51-89								51-89
	41-50								41-50
	≤ 40								≤ 40
URINE	Proteine								Proteine
	Glucosio								Glucosio
	Oligo-anuria*								Altro
RISPOSTA NEUROLOGICA	Allerta (A)								Allerta (A)
	Voce (V)								Voce (V)
	Dolore (P)								Dolore (P)
	Non responsiva (U)								Non responsiva (U)
	Totale riquadri gialli								Totale riquadri gialli
	Totale riquadri rossi								Totale riquadri rossi
	Iniziali operatore								Iniziali operatore

* diuresi oraria < 0.5 ml/h

ALLEGATO 2.

STRUMENTI PER LA DIAGNOSI DI SEPSI

SEQUENTIAL ORGAN FAILURE ASSESSMENT SCORE (SOFA score)

Setting di impiego:

- conferma del sospetto di sepsi in pazienti con infezione presunta/documentata e screening per sepsi (NEWS) positivo, **in Pronto Soccorso Centrale e nei reparti di area medica e chirurgica.**
- diagnosi di sepsi in pazienti con infezione presunta/documentata in **Unità di Terapia Intensiva** e nei reparti di **Rianimazione.**

Il SOFA è lo score di riferimento per la valutazione del danno d'organo a cui è affidata la diagnosi di sepsi.
Si compone di 6 distinte variabili, con singoli punteggi da 0 a 4, che misurano lo stato funzionale dei sistemi respiratorio, cardiovascolare, epatico, emocoagulativo, neurologico e renale.

SOFA score

FUNZIONE	0	1	2	3	4
**RESPIRATORIA** PaO_2/FiO_2	>400	≤400	≤300	≤200 in ventilazione meccanica	≤100 in ventilazione meccanica
**EMOCOAGULATIVA** PLT(conta/mm^3)	>150	≤150	≤100	≤50	≤20
EPATICA Bilirubina totale (mg/dL)	<1.2	1.2-1.9	2-5.9	6-11.9	>12
**CARDIOVASCOLARE**		PAM <70 mmHg	Dopamina ≤5μ/kg/min oppure Dobutamina	Dopamina >5μ/kg/min oppure Noradrenalina ≤0.1μ/kg/min	Dopamina >15μ/kg/min oppure Noradrenalina >0.1μ/kg/min
**NEUROLOGICA** GCS	15	13-14	10-12	6-9	< 6
**RENALE** Creatinina (mg/dl)/ Diuresi (ml/24 ore)		1.2-1.9	2-3.4	3.5-4.9 o < 500 ml/24 h	5 o < 200 ml/24 h

BOX 2. La misurazione del SOFA Score nella pratica clinica

Il **punteggio basale** del SOFA è assunto uguale a zero, a meno che il paziente non sia già noto avere una disfunzione d'organo antecedente all'episodio infettivo. La somma dei punteggi attribuiti ad ogni organo/apparato va da 0 a 24. La diagnosi di sepsi richiede un incremento acuto del SOFA score ≥ 2 punti e si associa ad un rischio di morte di circa il 10% nella popolazione generale ospedalizzata con sospetta infezione.

- Il **Rapporto PaO_2/FiO_2 (P/F)** correla la PaO_2 rilevata all'EGA con la FiO_2, ovvero con la percentuale di ossigeno nella miscela di gas inspirata dal paziente. Indica l'efficacia del passaggio di O_2 attraverso la membrana alveolo-capillare.
- Il **Glasgow Coma Scale (GCS)** prevede la valutazione combinata delle risposte oculari, verbali e motorie. Ad ognuna di queste corrisponde un punteggio la cui somma rappresenta lo score e definisce il livello di coscienza del paziente. Lo score risulta dalla somma della migliore risposta oculare, verbale e motoria ottenuta. La risposta motoria va testa a livello degli arti superiori. Uno stimolo algico appropriato e standardizzato è rappresentato dalla compressione del letto ungueale o da una pressione sullo sterno del paziente esercitata dall'operatore con le nocche delle dita.

Il punteggio dello score è compreso tra 3 e 15. Un GCS di 15 definisce un paziente cosciente e punteggi progressivamente più bassi indicano una compromissione crescente della coscienza fino al coma, che corrisponde a un valore di GCS ≤ 8.

Glasgow Coma Scale (GCS)

Parametro	Risposta	Punteggio
Apertura degli occhi	Spontanea	4
	Agli stimoli verbali	3
	Solo al dolore	2
	Nessuna risposta	1
Risposta verbale	Orientata e appropriata	5
	Confusa	4
	Con parole inappropriate	3
	Con parole incomprensibili	2
	Nessuna	1
Risposta motoria	Obbedisce al comando	6
	Localizza il dolore	5
	Retrae al dolore	4
	Flette gli arti al dolore	3
	Estende gli arti al dolore	2
	Nessuna risposta al dolore	1

SEQUENTIAL ORGAN FAILURE ASSESSMENT SCORE MODIFICATO (mSOFA score)

Setting di impiego:

- Conferma del sospetto di sepsi nelle pazienti ostetriche a rischio intermedio-alto secondo gli strumenti di screening, in Pronto Soccorso Ostetrico e nel reparto di Ostetricia.

SEQUENTIAL ORGAN FAILURE ASSESSMENT SCORE MODIFICATO (mSOFA score)

FUNZIONE	PARAMETRO	PUNTEGGIO 0	PUNTEGGIO 1
**Respiratoria**	Necessità di O_2 terapia per mantenere una $SpO_2 > 95\%$	No	Si
**Emocoagulativa**	Conta piastrinica/mm^3	> 100 000	< 100 000
**Epatica**	Bilirubina sierica (mg/dl)	< 1.2	> 1.2
**Cardiovascolare**	PAS (mmHg)<br>e/o<br>PAM (mmHg)	> 90<br><br>> 65	< 90<br><br>< 65
**Neurologica**	Scala AVPU	Sveglia (A)	Risvegliabile al richiamo verbale (V) o dopo stimolo doloroso (P) o incosciente (U)
**Renale**	Creatinina sierica, mg/dl	< 1.2	> 1.2

SpO_2 *saturazione periferica di ossigeno rilevata con saturimetro*; PAS *pressione arteriosa sistolica*; AVPU *Alert (sveglia), V verbal (risvegliabile al richiamo verbale), P pain (risvegliabile dopo stimolo doloroso), U unresponsive (incosciente).*

BOX 2. **LA MISURAZIONE DEL mSOFA SCORE NELLA PRATICA CLINICA.**

La versione modificata del SOFA score differisce dalla versione originaria nei seguenti punti:

- a ciascun parametro viene attribuito il punteggio 0 (valori fisiologici nella popolazione ostetrica) o 1 (valori patologici indicativi di possibile danno d'organo sepsi-indotto nella popolazione ostetrica). Pertanto, **in una paziente ostetrica con infezione certa/presunta, un**

mSOFA ≥ 1 sarà indicativo di danno d'organo e consentirà la diagnosi di sepsi. La scelta di un cut-off più basso rispetto alla popolazione generale (≥2) è giustificata dal rischio di un ritardo nella diagnosi e dalla rapidità di deterioramento clinico tipica di questa popolazione di pazienti.

- lo score definisce **valori soglia specifici per la popolazione ostetrica**. In questa popolazione, ad esempio, la creatininemia e la bilirubinemia sono fisiologicamente comprese tra 0.5 e 0.9 mg/dl. Pertanto, un valore di 1.2 mg/dl è da considerarsi patologico e potenzialmente indicativo di danno d'organo.

- lo score semplifica la **valutazione neurologica**, affidata al Glasgow Coma Score nella versione originaria. Nella versione modificata viene impiegato il **sistema AVPU**, già impiegato nel sistema MEOWS. Il GCS verrà valutato all'arrivo dell'equipe rianimatoria o dall'anestesista d'urgenza.

- la **valutazione iniziale del sistema respiratorio** è anch'essa semplificata, poiché il rapporto tra PaO_2 e FiO_2, che richiede l'esecuzione di una emogasanalisi e nello score originario è unico parametro di valutazione, può essere in prima battuta sostituito dal rilievo della **SpO_2** e dalla necessità o meno di ossigeno supplementare per mantenere una SpO_2 > 95%.

È opportuno sottolineare due aspetti:

- poiché l'ipotensione arteriosa può essere un riscontro fisiologico in gravidanza, il rilievo di una PAS < 90 mmHg o di una PAM < 65 mmHg deve essere interpretato alla luce dei valori abituali di pressione arteriosa della paziente;
- poiché la valutazione del mSOFA score può richiedere tempo necessario alla refertazione degli esami emato-chimici (dosaggio di bilirubinemia e creatininemia e determinazione della conta piastrinica), **in presenza di un fondato sospetto clinico e/o di un rischio intermedio o alto al MEOWS è opportuno procedere ai necessari interventi diagnostico-terapeutici.**

ALLEGATO 3.

CRITERI DI ALTO RISCHIO DI SEPSI NELLA POPOLAZIONE GENERALE E NELLA POPOLAZIONE OSTETRICA.

Tabella 4. **Categorie di pazienti ad alto rischio di infezione/sepsi nella popolazione generale.**

**Età**	Età < 1 anno o > 75 anni o condizioni di "fragilità"
**Immunodepressione franca**	Trapianto di organo solido
	Trapianto di cellule staminali emopoietiche
	Neoplasia maligna in chemio o immuno-terapia
	HIV con conta dei CD4 < 250/mm3
	Terapia con farmaci immunosoppressori
	Terapia con anticorpi monoclonali
**Alterata risposta immunologica alle infezioni**	Diabete mellito
	Etilismo cronico
	Anemia a cellule falciformi
	Asplenia anatomica o funzionale
	Uso cronico di steroidi (> 20 mg di prednisone o equivalenti per > 2 settimane negli ultimi 3 mesi)
**Alterata integrità della barriera cutaneo-mucosa**	Ferite, ustioni, infezioni cutanee
	Presenza di devices (catetere vescicale a permanenza, CVC, drenaggi)
	Uso di droghe per via iniettiva

*fragilità: sindrome biologica e clinica caratterizzata da riduzione della riserva funzionale e della resistenza agli stress, provocata dal declino cumulativo di più sistemi fisiologici in conseguenza di fattori biologici, psicologici e sociali (multiple e gravi comorbidità, invecchiamento avanzato con conseguente labilità omeostatica, alimentazione incongrua, polifarmacoterapia complessa, stato socio-ambientale critico, presenza di dipendenza funzionale, abuso alcolico o di fumo, istituzionalizzazione).

Tabella 5. **Categorie di pazienti ad alto rischio di infezione/sepsi nella popolazione ostetrica**.

CONDIZIONI MATERNE PRE-ESISTENTI LA GRAVIDANZA
**Fattori costituzionali e abitudini di vita**<br>❖ Età > 40 o < 18 anni.<br>❖ BMI > 30 kg/m2<br>❖ Fumo di sigaretta.<br>❖ Condizioni di deprivazione sociale
**Anamnesi**<br>❖ Diabete.<br>❖ Anemia grave (Hb < 9 g/dl)<br>❖ Malnutrizione<br>❖ Pregressa infezione da streptococco di gruppo B.<br>❖ Pregressa infezione pelvica<br>❖ Malattie immunologiche e/o uso di farmaci immunosoppressori.<br>❖ Stretto contatto con persone/familiari affetti da infezioni da streptococco di gruppo A.
**CONDIZIONI MATERNE LEGATE ALLA GRAVIDANZA**
❖ Assistenza ostetrica inadeguata.<br>❖ Infezioni da streptococco di gruppo A in soggetti a stretto contatto con la donna.<br>❖ Vaginosi batterica.<br>❖ Amniocentesi o altra procedura invasiva di diagnosi prenatale.<br>❖ Cerchiaggio cervicale.<br>❖ Rottura prolungata delle membrane amniocoriali.<br>❖ Induzione del travaglio.<br>❖ Secrezioni vaginali patologiche.<br>❖ Utilizzo di antibiotici nelle due settimane che precedono il parto.
**CONDIZIONI MATERNE LEGATE AL TRAVAGLIO E AL PARTO**
❖ Prolungamento della fase attiva del I stadio.<br>❖ Numero eccessivo (>5) di esplorazioni vaginali nel secondo stadio.<br>❖ Parto operativo.<br>❖ Parto pretermine<br>❖ Corion-amniosite<br>❖ Rimozione manuale della placenta.<br>❖ Taglio cesareo (soprattutto se non programmato o eseguito dopo l'inizio del travaglio)<br>❖ Episiotomia<br>❖ Emorragia precoce<br>❖ Ematomi e traumi perineali.<br>❖ Lacerazioni di III e IV grado.
**PERIODO POST-NATALE**
❖ Ritenzione di materiale placentare.<br>❖ Infezioni delle vie urinarie.

- Infezione della ferita chirurgica o e dell'episioraffia.
- Emorragia tardiva.
- Infezione del sito d'inserzione del catetere peridurale.
- Mastite.

ALLEGATO 4.

IL BUNDLE SEPSIS SIX MODIFICATO

1. SOMMINISTRAZIONE DI O2.

A livello cellulare la sepsi è caratterizzata da un'aumentata richiesta di O2, spesso associata a una sua ridotta disponibilità e/o a una ridotta capacità di estrazione dell'O2 stesso. Nel sospetto di sepsi e in presenza di un ridotto valore di SpO2 (< 94%), la tempestiva somministrazione di O2 ne migliora il trasporto e, in caso di sepsi materna previene anche l'ipossia fetale. Nei pazienti a rischio di ritenzione di CO2 (BPCO medio-grave, patologie neuro-muscolari), il target di SpO2 è 88-92%.

2. RIANIMAZIONE FLUIDICA.

Una rianimazione fluidica efficace, avviata al primo riscontro di ipotensione e/o tachicardia e/o di un livello di lattati > 2 mmol/l, è d'importanza cruciale nel contrastare il danno d'organo da ipoperfusione, con l'obiettivo di raggiungere e mantenere una PAM ≥ 65 mmHg, necessaria a prevenire o limitare il danno d'organo. Poiché il ripristino di un'adeguata pressione di perfusione agli organi vitali nel più breve tempo possibile è l'obiettivo primario della rianimazione fluidica, in caso d'ipotensione refrattaria a un adeguato reintegro volemico, passo successivo sarà l'avvio dell'infusione di **farmaci vasopressori**, in prima istanza **noradrenalina**, la cui maggior efficacia e sicurezza rispetto alla dopamina è oramai acclarata.
È acquisizione recente che il sovraccarico idrico aggravi la prognosi dei pazienti con sepsi quanto l'ipovolemia. Pertanto, è indispensabile che la rianimazione fluidica e la somministrazione di vasopressori avvengano sotto **monitoraggio emodinamico**, anche al fine di valutare la tolleranza ai fluidi, ovvero il rapporto tra volume di liquidi infusi e la comparsa di complicanze da disfunzione endoteliale, di cui uno dei più importanti e clinicamente dimostrabile è l'edema interstiziale. Le caratteristiche e l'invasività del monitoraggio emodinamico dipenderanno dal setting ospedaliero in cui il paziente è allocato.
Le **alterazioni emodinamiche che caratterizzano la gravidanza** influiscono sull'inquadramento clinico e sulla gestione della paziente con ipotensione o shock settico.
In particolare:

- Il fisiologico aumento della frequenza cardiaca e del volume plasmatico può ritardare l'identificazione dell'ipovolemia sepsi-indotta che, quando manifesta, esprime un deterioramento clinico avanzato. Questo sottolinea l'importanza della tempestività della stabilizzazione emodinamica con fluidi e, se necessario, con vasopressori.

o A partire dalla 20^ settimana di gestazione, l'utero gravidico può causare compressione aorto-cavale, riduzione del ritorno venoso al cuore e ipotensione. In tal caso, il **decubito laterale sinistro** eliminerà una potenziale concausa d'ipotensione.

o A causa dell'intensa vasocostrizione compensatoria cui va incontro la placenta in risposta all'ipotensione materna, spesso il riscontro di *condizioni fetali non rassicuranti* è il primo segno di ipoperfusione materna sepsi-indotta. Pertanto:

- in tutti i casi d'ipotensione o shock materni, che espongono al rischio d'ipossia e acidosi nel feto, è indispensabile la **valutazione immediata del benessere fetale**.
- la **valutazione del benessere fetale** deve essere sistematica in tutte le pazienti gravide con infezione sospetta o accertata, anche in assenza di instabilità emodinamica manifesta, perché **strumento di allerta di deterioramento clinico materno imminente**.
- il riscontro di *condizioni fetali non rassicuranti* e non altrimenti giustificabili, in assenza di infezione manifesta, impone la ricerca **di un'infezione o di una sepsi occulte**.

BOX 3. **Rianimazione fluidica e impiego dei vasopressori nella pratica clinica.**

- La rianimazione fluidica va eseguita attraverso due accessi venosi di grosso calibro (16 G-18 G) e avviata in caso di ipotensione (PAM < 65 mmHg) e/o tachicardia (FC > 95 bpm) e/o di un livello di lattati > 2 mmol/l. Si avvale dell'infusione di uno o più boli di cristalloidi di 5-7 ml/kg ciascuno, da somministrare massimo in 15 minuti e ripetibili, ove opportuno.
- Cristalloidi di scelta sono il ringer lattato o le soluzioni bilanciate. Per il rischio di acidosi ipercloremica, la soluzione fisiologica non è indicata.
- In questa fase il monitoraggio deve avvalersi di una combinazione di parametri clinici e di laboratorio (PAM, FC, refill capillare, sensorio, livello dei lattati, diuresi), prestando attenzione anche ai segni di eventuale imbibizione polmonare (dispnea, rantoli crepitanti, desaturazione).
- Il target è il raggiungimento di una PAM di almeno 65 mmHg e il reintegro volemico è definito adeguato dopo somministrazione di 30 ml/kg di fluidi nell'arco di 2-3 ore.
- Nel caso in cui, dopo adeguato reintegro volemico (30 ml/kg), la PAM si mantenga al di sotto dei 65 mmHg, sarà indispensabile avviare **l'infusione di noradrenalina** sotto monitoraggio ECG, richiedere l'intervento dei rianimatori. In caso di segni di sovraccarico idrico, sarà opportuno sospendere l'infusione di fluidi e avviare l'infusione di noradrenalina.
- Durante la fase di rianimazione fluidica è utile, ove disponibile, la **valutazione della responsività ai fluidi** che, a seconda del setting e delle competenze specialistiche a disposizione, potrà avvalersi del *passive leg rising*, dell'ecografia della VCI, dell'ecografia polmonare, dell'ecocardiografia, della *Pulse Pressure Variation* o dello *Stroke Volume Variation*.
- I farmaci vasopressori, inclusa la **noradrenalina**, vanno infusi in vena centrale. Tuttavia, in caso di grave ipotensione e nell'attesa dell'arrivo dell'equipe dei rianimatori, è possibile avviare l'infusione di noradrenalina in vena periferica, diluendone 3 fiale (2 mg di noradrenalina tartrato in ciascuna fiala, corrispondenti a 1 mg di noradrenalina) in 250 ml di soluzione glucosata al 5% (12 mcg di noradrenalina per ml) e infondendo la soluzione in pompa volumetrica seguendo le indicazioni riportate in tabella. L'accesso venoso in cui viene infusa la noradrenalina deve essere di grosso calibro (16 G, 18 G) e se ne deve assicurare la stabilità,

poiché in caso di stravaso il farmaco può provocare necrosi tissutale. Il dosaggio iniziale di noradrenalina dovrà essere di 0.05-0.1 mcg/kg/min e potrà essere aumentato ogni 5-10 min di 0.03-0.05 mcg/kg/min per volta se la PAM si mantiene < 65 mmHg.

- Qualora il dosaggio di noradrenalina richiesto sia troppo elevato (> 1 mcg/kg/min), andrà valutata l'opportunità di associarvi un secondo farmaco come **l'adrenalina o la vasopressina**.
- L'utilizzo di un agente inotropo come la **dobutamina** è indicato solo nei casi di accertata disfunzione miocardica.
- Il paziente che richieda il trasferimento o che sia già degente in un setting ad alta intensità di cure (Terapia Intensiva specialistica o Rianimazione) e che presenti shock settico o persistenza di ipotensione e/o di un livello di lattati > 2 mmol/l dopo adeguato reintegro volemico, andrà incontro ad una **fase di ottimizzazione emodinamica** il cui obiettivo è di assicurare un adeguato trasportato di O2 agli organi. In questa fase è raccomandato l'impiego di un monitoraggio emodinamico avanzato che includa, oltre che parametri clinici ed emodinamici di base, anche la valutazione della funzione cardiaca, delle resistenze vascolari periferiche, della responsività ai fluidi o di un eventuale sovraccarico idrico mediante termodiluizione transpolmonare e monitoraggio della $ScvO_2$.

Tabella 6. **Velocità di infusione della noradrenalina in vena periferica in base al peso corporeo del paziente.**

DOSE (mcg/kg/min)	VELOCITA' DI INFUSIONE				
	50 kg	60 kg	70 kg	80 kg	90 kg
0,05	12 ml/h	15 ml/h	18 ml/h	20 ml/h	22,5 ml/h
0,1	25 ml/h	30 ml/h	35 ml/h	40 ml/h	45 ml/h
0,15	37,5 ml/h	45 ml/h	52,5 ml/h	60 ml/h	67,5 ml/h
0,20	50 ml/h	60 ml/h	70 ml/h	80 ml/h	90 ml/h
0,30	75 ml/h	90 ml/h	105 ml/h	120 ml/h	135 ml/h
0,40	100 ml/h	120 ml/h	140 ml/h	160 ml/h	180 ml/h
3 fiale di noradrenalina tartrato da 2 mg (ciascuna corrispondente ad 1 mg di noradrenalina), diluite in 250 ml di soluzione glucosata al 5% (12 mcg/ml di noradrenalina).					

3. **MONITORAGGIO DELLA DIURESI ORARIA.**

Va avviato il prima possibile. La presenza di oliguria (< 0.5 ml/kg/h) o anuria è segno di compromissione emodinamica o di danno renale acuto sepsi-indotti. Il monitoraggio orario consente di valutare la risposta emodinamica alla terapia.

4. **PRELIEVO PER EMOCOLTURA E COLTURE DA SITI SOSPETTI.**

Va eseguito sempre prima della somministrazione della terapia antibiotica, ma senza che questa subisca un sostanziale ritardo. Per la potenziale rapidità evolutiva della sepsi e dello shock settico, la terapia antimicrobica empirica deve per necessità essere ad ampio spettro. L'identificazione dell'agente eziologico dell'infezione e la definizione del suo profilo di sensibilità agli antimicrobici mediante emocoltura e colture da siti sospetti (liquor in caso di meningite sospetta, urine in caso di sospetta urosepsi, espettorato o aspirato tracheo-bronchiale o BAL in caso di infezione delle vie respiratorie) è fondamentale a fini prognostici, in quanto consente a posteriori la **verifica dell'appropriatezza della terapia** antibiotica empirica avviata e, spesso, la sua semplificazione, limitando la selezione di patogeni multi-resistenti agli antibiotici, gli eventi avversi e il dispendio di risorse economiche. Per l'alta probabilità che gli esami colturali risultino negativi, è indispensabile che il prelievo dei campioni avvenga prima della somministrazione della terapia empirica, avendo cura che non ne ritardi eccessivamente l'avvio. La diagnostica molecolare rapida (ricerca dell'agente eziologico mediante real time PCR) e la ricerca di antigeni urinari di legionella e pneumococco non risentono dell'effetto degli antibiotici. Pertanto, laddove indicate, possono essere avviate anche subito la somministrazione della terapia empirica.

BOX 4. **Emocoltura nella pratica clinico-assistenziale.**

- Il prelievo deve essere eseguito immediatamente, alla diagnosi o al sospetto clinico di sepsi, senza attendere la comparsa di brivido o il picco febbrile.

- Il prelievo deve essere effettuato prima dell'avvio della terapia antibiotica empirica, ma non deve in alcun modo ritardarlo. Se la terapia antibiotica è già in corso, può essere utile ripetere le emocolture prima della somministrazione della dose successiva.

- Il prelievo deve essere effettuato preferibilmente per venipuntura da vena periferica. Se è in sede un catetere venoso centrale da più di 48 ore, uno dei due prelievi va effettuato da un lume di quest'ultimo, l'altro da venipuntura. In caso di CVC multilume, se possibile, andrà utilizzato un set per ciascun lume.

- Il prelievo va eseguito rispettando i seguenti steps:

a. eseguire un'accurata **antisepsi delle mani** mediante frizione con soluzione di CHG 0.5% in alcol 70% (*Esoform mani cute*®) o lavaggio con soluzione saponosa (liquida o caricata in spugne monouso) di uno dei seguenti antisettici ad attività protratta (in ordine decrescente di preferenza): CHG 4% (Neo Sterixidina Soap 4%® ovvero *Spazzolino Chir con Spugna Clorexidina 5% Monouso*), CHG 2% (*Dermosept*®), PVI ≥ 5% (*Braunol 7,5%*® sol. cutanea con tensioattivo), **secondo la sequenza OMS.**

b. indossare guanti sterili e i dispositivi di barriera come indicato dalle precauzioni standard;

c. effettuare l'antisepsi con clorexidina gluconato al 2% in alcool isopropilico al 70% (in alternativa, iodopovidone al 10% in soluzione alcolica);

d. rispettare i tempi di contatto (30 secondi per la clorexidina e oltre i 2 minuti per lo iodopovidone) del prodotto sulla cute ed attendere che si asciughi naturalmente;

f. eseguire il prelievo con tecnica asettica;

g. disinfettare il diaframma di gomma dei flaconi con etanolo al 70% o alcool isopropilico e lasciarlo asciugare prima di inoculare il sangue.

i. eseguire un accurata igiene delle mani con soluzione alcolica di clorexidina gluconato o sapone antisettico, secondo la sequenza OMS.

- Il volume di sangue prelevato è la variabile più significativa del test. È necessario l'invio di almeno due set (1 set è costituito da un flacone per aerobi e da un flacone per anaerobi), in ciascuno dei quali vanno inseriti 8-10 ml di sangue. Il mantenimento dei flaconi in posizione verticale rappresenta un utile metodo per assicurare che venga inoculata la giusta quantità di sangue. Se si utilizza un sistema vacutainer®, è necessario prima riempire il flacone per aerobi e poi quello per anaerobi, per evitare che l'aria presente nel raccordo venga introdotta nel campione.

- La consuetudine di far trascorrere 30 minuti tra un prelievo e l'altro è arbitraria. I due set possono anche essere raccolti consecutivamente. Solo in caso di sospetta endocardite, è preferibile effettuare i prelievi a distanza di 30-60 minuti (per documentare la batteriemia continua) e, in caso di negatività, inviare altri 3 set dopo 24h.

- L'invio in laboratorio deve essere immediato. I campioni andranno inviati al laboratorio di microbiologia dalle 8.00 alle 13.00, dal lunedì al sabato, al laboratorio d'urgenza dalle 13.00 alle 07.00, dal lunedì al sabato e a qualsiasi ora la domenica. Corrette modalità di prelievo, conservazione ed invio dei campioni microbiologici sono essenziali per garantire risultati attendibili.

5. PRELIEVO PER ESAMI EMATOCHIMICI.

EGA e misura dei lattati. In corso di sepsi, il livello dei lattati è un indice surrogato d'ipoperfusione d'organo. In pazienti ad alto rischio di deterioramento clinico (screening positivo) o con sepsi, il livello di lattati consente di stratificare l'intensità del trattamento da adottare. Un livello di lattati > 2 mmol/l definisce ad alto rischio un paziente con infezione e, in presenza di ipotensione refrattaria al reintegro volemico che richieda l'impiego di vasopressori per mantenere una PAM ≥ 65 mmHg, sancisce la diagnosi di shock settico.
Durante la rianimazione fluidica, l'andamento temporale del livello dei lattati è una misura della risposta alla terapia: un aumento dei lattati indica un elevato rischio di morte e la necessità di revisione immediata della strategia terapeutica. Oltre che sul livello dei lattati, l'emogas-analisi fornisce, inoltre, preziose informazioni sull'equilibrio acido-base e sugli scambi gassosi dei pazienti.

BOX 5. **MISURA DEI LATTATI**

- Per la necessità di disporne in tempi rapidi (15 minuti al massimo), la **misura dei lattati** deve essere eseguita mediante emogasanalisi e non richiesta al laboratorio centrale. In caso di difficoltà nell'eseguire un prelievo di sangue arterioso, il dosaggio è egualmente affidabile se eseguito su sangue venoso.
- In caso di riscontro di un livello di lattati > 2 mmol/l è necessario ripetere il dosaggio dopo 1-2 ore dall'inizio della rianimazione fluidica. In caso di valori persistentemente al di sopra di 2 mmol/l, il monitoraggio va proseguito almeno ogni 2 ore.

Pannello sepsi. Comprende la misura degli indici di flogosi, dei biomarcatori d'infezione (PCT, PSEP, Pro-Adm) e degli indici di funzione/danno d'organo necessari per il calcolo del SOFA e del mSOFA score. È dunque di ausilio alla conferma diagnostica d'infezione e indispensabile per la diagnosi di sepsi.
Ad oggi, nessun biomarcatore è stato validato per la diagnosi di sepsi. Tuttavia, nella pratica clinica è noto che sia la loro determinazione all'esordio della sepsi, che il loro monitoraggio in corso di terapia possono fornire preziose informazioni sulla evoluzione clinica dei pazienti. Poiché ciascun biomarcatore ha proprie pecularità, l'approccio oggi suggerito nella gestione del paziente con infezione/sepsi è quello multi-marker. I biomarcatori di infezione (PCT, PSEP e ProAdm) consentono una stratificazione prognostica dei pazienti con sospetta sepsi e la titolazione dell'intensità delle cure richieste, sono di supporto alla diagnosi di infezione e una guida sulla possibile eziologia della stessa (PCT), possono anticipare l'insorgenza di disfunzioni d'organo in pazienti con infezione (ProAdm). Inoltre, il trend temporale dei biomarcatori riflette l'andamento clinico della sepsi ed è di supporto alla sospensione della terapia antimicrobica. È dimostrato che la terapia antibiotica può essere sospesa senza alcuna ripercussione negativa in caso di una evoluzione clinica favorevole, associata ad una riduzione del valore di PCT del 80% rispetto al suo valore al picco o una sua riduzione al di sotto di 0.5 mcg/l.

BOX 6. **PANNELLO SEPSI**

Comprende la misura di:

- emocromo con formula leucocitaria
- proteina C reattiva, procalcitonina, presepsina, pro-adrenomedullina.
- creatininemia
- bilirubinemia, transaminasi (AST, ALT)
- elettroliti sierici (Na, K, Ca, Mg)
- PT, PTT, fibrinogeno, d-dimeri.

Il pannello sepsi, esclusa la procalcitonina, andrà avviato presso il laboratorio d'urgenza, disponibile sul sistema Galileo. Poiché non disponibile in urgenza, la procalcitonina andrà dosata in routine, nel minor tempo possibile dal sospetto clinico di infezione.

6. TEMPESTIVA SOMMINISTRAZIONE DI UN REGIME DI TERAPIA EMPIRICA AD AMPIO SPETTRO.

Nello shock settico, ogni ora di ritardo nella somministrazione della terapia antibiotica rispetto all'insorgenza dell'ipotensione comporta un sostanziale incremento della mortalità. Pertanto, la SSC raccomanda che in presenza di **sepsi o shock settico** presunti o certi, si provveda **il prima possibile e comunque entro la prima ora** dalla diagnosi alla somministrazione per via endovenosa di uno o più farmaci antibiotici ad ampio spettro. Evidenze più recenti rimarcano l'importanza di tale approccio **solo nello shock settico**, ove è dimostrato che una rigorosa tempestività influenza direttamente la mortalità. In assenza di shock, una tempistica più conservativa, in attesa dei risultati microbiologici, migliorerebbe l'appropriatezza terapeutica, garantendo benefici sia sull'outcome del singolo paziente che sull'epidemiologia locale.
Tale approccio presuppone la disponibilità continuativa di metodiche di microbiologia rapida che consentano l'identificazione dell'agente eziologico dell'infezione e dei suoi principali meccanismi di resistenza nell'arco di poche ore. Laddove non si disponga di tali metodiche, la somministrazione degli antibiotici dovrà avvenire entro la prima ora dal sospetto clinico, sia di sepsi che di shock settico.

BOX 7. **CRITERI DI SCELTA DELLA TERAPIA ANTIBIOTICA EMPIRICA**

La terapia empirica va avviata il più rapidamente possibile, con farmaci ad ampio spettro, dopo esecuzione delle emocolture e di eventuali altri esami colturali da siti sospetti.

La scelta del regime antibiotico deve tener conto del sito d'infezione, che condiziona i probabili patogeni coinvolti, dell'epidemiologia locale, della diffusibilità delle molecole a livello del sito di infezione e della presenza di fattori di rischio per patogeni multi-resistenti agli antibiotici.

Durante il percorso nascita, la scelta del regime antibiotico deve avvenire nella consapevolezza delle classi FDA di sicurezza materno-fetale delle molecole antimicrobiche (tab. 7).

Al momento della refertazione degli esami colturali, è indispensabile procedere, con l'ausilio del consulente infettivologo, alla rimodulazione della terapia antibiotica (**de-escalation**), alla luce della risposta clinica e del trend della PCT.

In seconda o terza giornata, sulla base del patogeno isolato e del suo profilo di sensibilità agli antimicrobici, il regime di terapia empirica adottato andrà rimodulato (**de-escalation**), mediante sospensione dei farmaci non necessari e prosecuzione della terapia con la molecola a spettro più ristretto e a maggiore attività nei confronti dell'isolato. Estrema attenzione andrà posta all'impiego dei carbapenemici solo quando indispensabile e alla sostituzione di vancomicina, teicoplanina e daptomicina con cefazolina o oxacillina in caso di isolamento di stafilococchi sensibili alla meticillina.

La **durata** della terapia antibiotica sarà dettata dall'evoluzione clinica e dal trend dei biomarcatori.

Tabella 7. **Fattori di rischio per patogeni "difficili"**

Enterobatteri produttori di ESBL	Pregressa colonizzazione e/o infezione (ultimi 12 mesi)<br>Prolungata ospedalizzazione<br>Multipli cicli di terapia antibiotica nell'ultimo mese<br>Catetere vescicale a permanenza o PEG
Pseudomonas aeruginosa	Pregressa colonizzazione e/o infezione (ultimi 12 mesi)<br>Multipli cicli di terapia antibiotica nell'ultimo mese<br>Alterazioni strutturali polmonari (bronchiectasie, BPCO grave, fibrosi cistica, ostruzione bronchiale).<br>Diabete mellito complicato<br>Catetere vescicale a permanenza<br>Età avanzata<br>Etilismo<br>Immunocompromissione*
Stafilococco aureo resistente alla meticillina (MRSA)	Pregressa colonizzazione e/o infezione (ultimi 12 mesi)<br>Emodialisi, dialisi peritoneale<br>CVC in sede da > 48 ore o catetere vascolare a permanenza<br>Cicli multipli di terapia antibiotica<br>Residenza in lungodegenza o recente ospedalizzazione (ultimi 3 mesi)

	Immunocompromissione*
Candida *spp*	Recente infezione o colonizzazione multifocale<br>Chirurgia addominale maggiore/pancreatite necrotizzante<br>Gravi comorbidità (diabete mellito, insufficienza renale o epatica cronica).<br>Catetere vascolare a permanenza<br>Nutrizione parenterale

* **Immunocompromissione:** HIV con CD4+ < $200/mm^3$, trapianto di midollo e/o di organo solido, chemioterapia negli ultimi 2 mesi, terapia steroidea (prednisone 20 mg/die o equivalenti) da almeno 4 settimane, neutropenia persistente (< 500 mm^3).

TERAPIA ANTIMICROBICA EMPIRICA PER SITO DI INFEZIONE

POLMONITE

A. Setting. Una tra le seguenti condizioni:

- Sepsi ad esordio in comunità.

- Sepsi ad esordio in ospedale o altra struttura assistenziale in paziente ricoverato da ≤ 48 ore.

Assenza di fattori di rischio per patogeni MDR o per *P. aeruginosa.**

Ceftriaxone + [claritromicina o azitromicina].

Se allergia a beta-lattamici: levofloxacina

- Rischio *P. aeruginosa:**

[piperacillina-tazobactam o cefepime o ceftazidime] + levofloxacina + [claritromicina o azitromicina].

Se allergia a beta-lattamici: meropenem + levofloxacina.

- Rischio MRSA*:

+ [linezolid o vancomicina] *oppure* ceftobiprole + [claritromicina o azitromicina].

Se allergia a beta-lattamici: linezolid + claritromicina.

- Rischio ESBL*:

meropenem + claritromicina

- Stagione influenzale: + oseltamivir.

B. Setting. Una tra le seguenti condizioni:

- Shock settico

- Sepsi ad esordio in ospedale o in altre strutture assistenziali in paziente ricoverato da > 48 ore.

- Sepsi in presenza di rischio di infezione da patogeni MDR.

- Assenza di shock settico, rischio ESBL, colonizzazione/pregressa infezione da MDR*:

[Piperacillina-tazobactam o cefepime] + levofloxacina + [linezolid o vancomicina].

Se allergia a beta-lattamici: meropenem + [linezolid o vancomicina].

- **Shock settico**:

meropenem + [linezolid o vancomicina] + [claritromicina o azitromicina].

- Rischio ESBL*: meropenem + [claritromicina o azitromicina].

- Colonizzazione/pregressa infezione da *K. pneumoniae* KPC:

[ceftazidime-avibactam** o colistina ev + aerosol] + [meropenem o fosfomicina] + [linezolid o vancomicina].

- Colonizzazione/pregressa infezione da P. aeruginosa MDR:

ceftolozane-tazobactam** o colistina + fosfomicina + [linezolid o vancomicina].

- Colonizzazione/pregressa infezione da A. baumannii MDR:

[colistina ev + aerosol] + ampicillina-sulbactam + meropenem + [linezolid o vancomicina].

- Impegno interstiziale polmonare: + claritromicina o azitromicina.

*** vedi tabella 6. ** necessaria consulenza infettivologica.**

DIAGNOSTICA MICROBIOLOGICA.

Prima dell'avvio della terapia antibiotica, ma senza che questa subisca un sostanziale ritardo, avviare:

- emocolture
- esame colturale su campione respiratorio (espettorato, secreto bronchiale, BAL) con ricerca di germi comuni e miceti, presso il laboratorio di microbiologia.
- esame colturale su campione respiratorio per ricerca di legionella, presso il laboratorio di microbiologia.
- Sierologia per patogeni respiratori presso il laboratorio di microbiologia.
- ricerca antigeni urinari di legionella e pneumococco, presso il laboratorio di microbiologia.

- pannello patogeni respiratori in real time PCR, presso il laboratorio di Igiene.
- ricerca virus influenza A e B se stagione invernale presso il laboratorio di Igiene.

INFEZIONE DELLE VIE URINARIE

A. Setting. Una tra le seguenti condizioni:

- Sepsi ad esordio in comunità.

- Sepsi ad esordio in ospedale o in altre strutture assistenziali in paziente ricoverato da ≤ 48 ore.

Piperacillina-tazobactam + amikacina.

Se allergia a beta-lattamici: ciprofloxacina + gentamicina.

B. Setting. Una tra le seguenti condizioni:

- Shock settico

- Sepsi ad esordio in ospedale o in altre strutture assistenziali in paziente ricoverato da > 48 ore.

- Sepsi in presenza di rischio di infezione da patogeni MDR.

- Assenza di shock settico o di colonizzazione/pregressa infezione da MDR.

Meropenem + amikacina.

- Colonizzazione/pregressa infezione da K. pneumoniae KPC:

[ceftazidime-avibactam** o colistina] + [meropenem o fosfomicina].

- Colonizzazione/pregressa infezione da P. aeruginosa MDR:

[ceftolozane-tazobactam** o colistina] + [fosfomicina o amikacina].

- Colonizzazione/pregressa infezione da A. baumannii MDR:

colistina + ampicillina-sulbactam + meropenem.

- Shock settico: + linezolid o vancomicina.

*** vedi tabella 6. ** necessaria consulenza infettivologica**

DIAGNOSTICA MICROBIOLOGICA.

Prima dell'avvio della terapia antibiotica, ma senza che questa subisca un sostanziale ritardo, avviare:

- **emocolture**
- **esame chimico-fisico urine**
- **urinocoltura**

INFEZIONE INTRA-ADDOMINALE

A. Setting. Una tra le seguenti condizioni:

- Sepsi ad esordio in comunità.

- Sepsi ad esordio in ospedale o in altre strutture assistenziali in paziente ricoverato da ≤ 48 ore.

Piperacillina-tazobactam oppure cefepime + metronidazolo.

Se allergia a beta-lattamici: ciprofloxacina + gentamicina + metronidazolo.

B. Setting. Una tra le seguenti condizioni:

- Shock settico

- Sepsi ad esordio in ospedale o in altre strutture assistenziali in paziente ricoverato da > 48 ore.

- Sepsi in presenza di rischio di infezione da patogeni MDR.

- Assenza di shock settico o di colonizzazione/pregressa infezione da MDR.

Imipenem o meropenem.

- colonizzazione/pregressa infezione da K. pneumoniae KPC:

[ceftazidime-avibactam** + metronidazolo] *oppure* colistina + fosfomicina o gentamicina ± tigeciclina.

- colonizzazione/pregressa infezione da P. aeruginosa MDR:

[ceftolozane-tazobactam** + metronidazolo] *oppure* colistina + [fosfomicina o amikacina].

- **shock settico**: + [daptomicina o linezolid o vancomicina].

- **shock settico o rischio di candidiasi invasiva***:

+ [anidulafungina o caspofungina o micafungina].

*** vedi tabella 6. ** necessaria consulenza infettivologica**

DIAGNOSTICA MICROBIOLOGICA.

Prima dell'avvio della terapia antibiotica, ma senza che questa subisca un sostanziale ritardo, avviare le emocolture.

È indispensabile procedere al prelievo di campioni intra-operatori da inviare in laboratorio per esame colturale in caso di intervento chirurgico

MENINGITE COMUNITARIA

A. A LIQUOR TORBIDO

ceftriaxone + vancomicina + desametasone (8 mg/6-8 ore per 2-4 giorni)

- fattori di rischio per Listeria*: + ampicillina.

A. A LIQUOR LIMPIDO

ceftriaxone + acyclovir

- fattori di rischio per Listeria*: + ampicillina

C. RACHICENTESI NON ESEGUIBILE IN TEMPI RAPIDI (GCS ≤ 8)

ceftriaxone + vancomicina + acyclovir + desametasone (8 mg/6-8 ore per 2-4 giorni).

fattori di rischio per Listeria*: + ampicillina

CORION-AMNIOSITE, ENDOMETRITE O ABORTO SETTICO.

A. SEPSI

- Assenza di fattori di rischio per ESBL o MRSA

Piperacillina/tazobactam + [Gentamicina o Amikacina].

- Rischio ESBL*:

Meropenem + [Gentamicina o Amikacina].
**- Rischio MRSA*:**
+ [Daptomicina o Vancomicina o Linezolid].

* Vedi tabella 6.

DIAGNOSTICA MICROBIOLOGICA.

Prima dell'avvio della terapia antibiotica, ma senza che questa subisca un sostanziale ritardo, avviare:

- Emocolture
- urinocoltura,
- tamponi cervico-vaginali con ricerca di germi comuni e mycoplasma
- tamponi rettali con ricerca dello streptococco beta emolitico gruppo B.
- L'amniocentesi con coltura del liquido amniotico è indicata nel sospetto di corion-amniosite subclinica.

**INFEZIONE DI CUTE E TESSUTI MOLLI**
**- Assenza di fattori di rischio per P. aeruginosa e MRSA.**
Amoxicilina-clavulanato + clindamicina.
**- Rischio P. aeruginosa*:**
Piperacillina-tazobactam + clindamicina.
**- Rischio MRSA***: ceftarolina + clindamicina.
Se allergia ai beta-lattamici: ciprofloxacina + clindamicina.

* Vedi tabella 6.

**SEPSI O SHOCK SETTICO DA FONTE NON NOTA**
**A.** Una tra le seguenti condizioni:
- Sepsi ad esordio in comunità.
- Sepsi ad esordio in ospedale o in altre strutture assistenziali in paziente ricoverato da $\leq$ 48 ore.
Piperacillina-tazobactam + amikacina + vancomicina

B. Una tra le seguenti condizioni:

- Shock settico

- Sepsi ad esordio in ospedale o in altre strutture assistenziali in paziente ricoverato da > 48 ore.

- Sepsi in presenza di rischio di infezione da patogeni MDR.

- Assenza di fattori di rischio per candidiasi invasiva* e di colonizzazione/pregressa infezione da patogeni MDR

Meropenem + linezolid o vancomicina

- rischio di candidiasi invasiva*:

+ [anidulafungina o caspofungina o micafungina].

- colonizzazione/pregressa infezione da K. pneumoniae KPC:

+ ceftazidime-avibactam.

- colonizzazione/pregressa infezione da P. aeruginosa MDR:

+ colistina + amikacina.

- colonizzazione/pregressa infezione da A. baumannii MDR:

+ colistina + ampicillina-sulbactam

* Vedi tabella 6.

Sicurezza dei farmaci antimicrobici in gravidanza: classificazione FDA.

CLASSE FDA	FARMACO
**CLASSE B: farmaci "sicuri"**	Acyclovir<br>Cefalosporine<br>Carbapenemi (tranne imipenem)<br>Daptomicina<br>Fosfomicina<br>Metronidazolo (evitare nel primo trimestre)<br>Nitrofurantoina<br>Penicilline
**CLASSE C: è richiesta una giudiziosa stima del rapporto rischi/benefici. Da impiegare con cautela, solo quando i potenziali benefici superano i rischi**	Caspofungina<br>Claritromicina<br>Fluconazolo<br>Fluorochinoloni<br>Imipenem-cilastatina<br>Linezolid

	Rifampicina<br>Trimethropim-sulfametossazolo<br>Vancomicina
**CLASSE D: potenzialmente nocivi. Da impiegare in situazioni di emergenza e a "rischio-vita", in assenza di alternative terapeutiche più sicure.**	Aminoglicosidi<br>Tigeciclina<br>Voriconazolo
**CLASSE X: controindicati in gravidanza**	Ribavirina

- o **Antimicrobici di Categoria B**: nessun rischio per il feto osservato in studi animali, ma non vi sono studi metodologicamente validati e controllati in donne in gravidanza OPPURE un evento avverso è dimostrato in studi animali, ma studi ben controllati in donne in gravidanza non hanno mostrato effetti nocivi per il feto in qualunque trimestre di gravidanza.
- o **Antimicrobici di Categoria C**: studi animali hanno mostrato tossicità per il feto, ma non vi sono studi metodologicamente validati e controllati condotti in donne in gravidanza; tuttavia, i loro potenziali benefici potrebbero giustificarne l'utilizzo in gravidanza, nonostante i potenziali rischi per il feto.
- o **Antimicrobici di Categoria D**: studi sull'uomo e dati di farmacovigilanza hanno evidenziato un rischio per il feto. Tuttavia, i loro potenziali benefici potrebbero giustificarne l'utilizzo in gravidanza, nonostante i potenziali rischi per il feto.
- o **Antimicrobici di Categoria X.** Studi sperimentali o clinici hanno dimostrato tossicità sul feto. I rischi associati all'uso di questi farmaci superano di gran lunga i benefici. Controindicati in gravidanza.

Avviato il bundle, sarà indispensabile procedere a:

7. **IDENTIFICAZIONE E CONTROLLO DELLA FONTE DI INFEZIONE.**

Rapido avvio delle indagini diagnostiche volte ad identificare o escludere rapidamente focolai infettivi eradicabili chirurgicamente o rimovibili, misura **da cui dipende il successo dell'intero trattamento.**

L'anamnesi e l'esame clinico indirizzeranno il clinico sulla diagnostica più idonea da avviare per la ricerca del focus.

Per "controllo della fonte" si intendono tutte le procedure disponibili per eliminare la fonte di infezione, ridurre l'inoculo batterico e correggere alterazioni anatomiche al fine di ripristinare le funzioni fisiologiche". Consiste nel drenaggio di ascessi (empiemi), asportazione di tessuto necrotico infetto (fasciite necrotizzante) o di corpi estranei infetti (CVC, catetere vescicale) e nella definitiva eliminazione della causa della contaminazione (ad. es perforazione intestinale). In assenza di una fonte nota di infezione, gli **accessi vascolari** (CVC, cateteri arteriosi) a breve termine presenti da > 48 ore andrebbero sostituiti. Per gli accessi a lungo termine, la rimozione va ponderata valutandone rischi e benefici. In caso di candidemia nota, il dispositivo va sempre rimosso.

Le misure di **controllo e/o eradicazione della fonte** di infezione dovranno essere espletate il prima possibile dopo la fase di rianimazione fluidica e comunque **entro 6-12 ore dalla diagnosi** di sepsi/shock settico, **impiegando, a parità di efficacia, la tecnica meno invasiva, con minor impatto sistemico, dopo attenta analisi del rapporto rischi/benefici.**

8. **Profilassi del trombo-embolismo venoso durante il percorso nascita.** Per una concomitanza di eventi che caratterizzano il binomio sepsi e gravidanza (stato pro-infiammatorio, attivazione della coagulazione o coagulazione intravascolare disseminata, stasi venosa, immobilità), nella popolazione ostetrica la sepsi espone a un rischio aggiuntivo di complicanze trombo-emboliche. In queste pazienti sarà pertanto indispensabile procedere alla profilassi meccanica e farmacologica della trombosi venosa profonda.

2. KAPLAN-MEIER PLOT

Kaplan-Meier plot of the probability of survival from enrollment to day 30

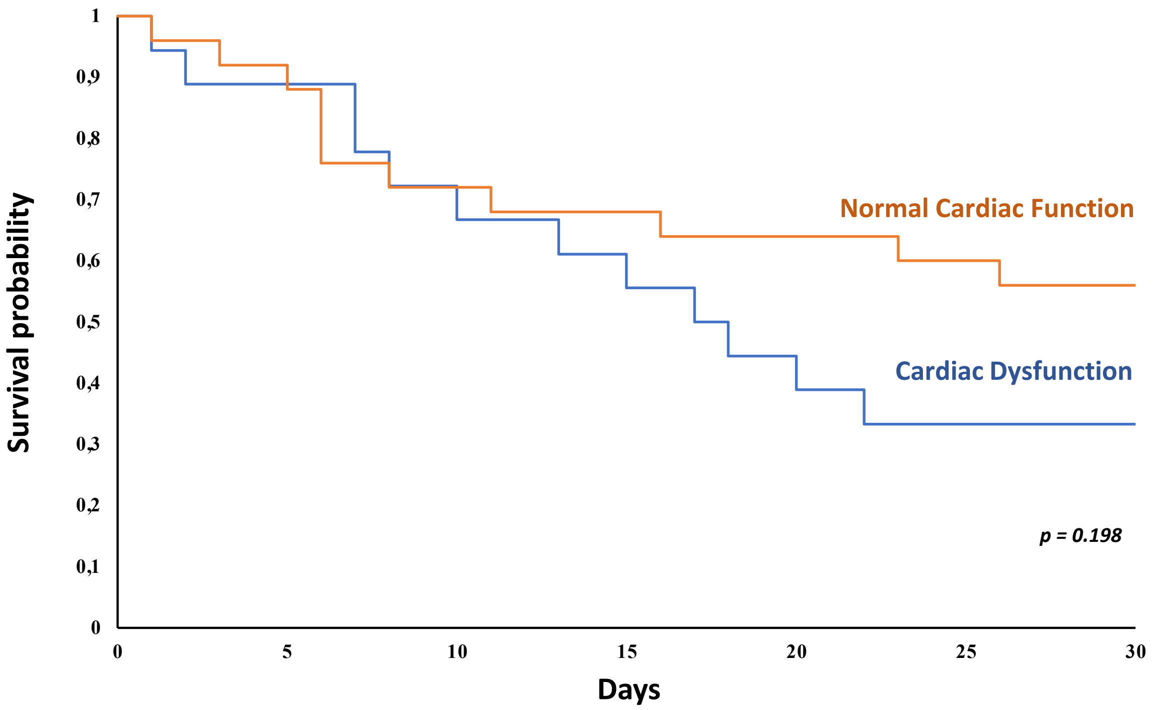

Supplement: Supplementary file 1 — Additional file 1. Online supplement. Subclinical cardiac dysfunction may impact on fluid and vasopressor administration during early resuscitation of septic shock. [file 44158_2023_117_MOESM1_ESM.pdf]
